# Screening of Rhizobacteria Capable of Modulating Cd Accumulation and Enhancing Pharmacopoeial Constituents in *Salvia miltiorrhiza*

**DOI:** 10.3390/plants15182858

**Published:** 2026-09-18

**Authors:** Jinqiu Liao, Tong Peng, Zheng’e Han, Kecen Pan, Meishen Liu, Li Zhang, Ruiwu Yang

**Affiliations:** 1 College of Life Sciences, Sichuan Agricultural University, Ya’an 625014, China; liaojinqiu630@sicau.edu.cn (J.L.); pt18280798208@sina.com (T.P.); hanzhenge2023@163.com (Z.H.); pkc0089@sina.com (K.P.); 18283567896@163.com (M.L.); 2 College of Science, Sichuan Agricultural University, Ya’an 625014, China; zhangli@sicau.edu.cn

**Keywords:** Cd-tolerant bacteria, Cd bioavailability, cell wall, pectin, hemicellulose

## Abstract

Cadmium (Cd) contamination poses a severe threat to the quality of cultivated land and the safe production of food crops and Chinese herbal medicines in China. The Cd accumulation characteristics of *Salvia miltiorrhiza* are closely associated with rhizosphere microorganisms; however, the underlying regulatory mechanisms remain elusive. In this study, two *S. miltiorrhiza* genotypes with high or low Cd accumulation (designated SD and SC, respectively) were employed to comparatively analyze the differences in their rhizosphere bacterial community structures. Combined with reinoculation experiments using bacterial strains tolerant to Cd, we aimed to elucidate the mechanistic roles of rhizosphere bacteria in modulating Cd accumulation in *S. miltiorrhiza*. 16S rRNA sequencing revealed significant differences in the rhizosphere bacterial community structures between SD and SC, with SC exhibiting higher bacterial diversity and richness, confirming a correlation between rhizosphere microbial community composition and Cd accumulation in *S. miltiorrhiza*. Functional validation demonstrated that strain *Bacillus* sp. C4, isolated from SC, significantly reduced Cd accumulation in *S. miltiorrhiza* roots by 20.35%, whereas strain *Bacillus* sp. D9, isolated from SD, exerted a contrasting effect, drastically increasing root Cd accumulation by 440.97%. Two distinct rhizobacterial strains were identified with contrasting strategies for modulating Cd partitioning in the soil root continuum of *S. miltiorrhiza*. Strain C4 acts as a Cd excluder: it elevates rhizosphere pH and adsorbs Cd externally, while decreasing pectin and hemicellulose contents in root cell walls to reduce Cd binding sites, collectively impeding Cd entry into roots. In contrast, strain D9 functions as a Cd accumulator through a dual mechanism. It acidifies the rhizosphere to mobilize sparingly soluble Cd, thereby increasing Cd influx. Concurrently, it upregulates hemicellulose abundance in root cell walls, which amplifies Cd binding capacity and promotes root sequestration. Furthermore, inoculation with strain C4 significantly elevated the contents of bioactive components in *S. miltiorrhiza*, including cryptotanshinone, tanshinone I, tanshinone IIA, and salvianolic acid B, demonstrating a dual beneficial functionality of concurrent Cd reduction and quality improvement. This study offers functional strains that reduce Cd accumulation and enhance pharmacopoeial constituents of *S. miltiorrhiza*, supporting the production of low-Cd, high-quality medicinal materials in Cd-contaminated farmland.

## 1. Introduction

With the acceleration of industrialization and intensive agriculture, human activities including mineral smelting, industrial emissions, and the excessive application of agricultural chemicals, together with certain natural soil formation processes, have made cadmium (Cd) contamination an increasingly severe problem in China’s soils. As one of the most biologically toxic heavy metals, Cd can accumulate progressively through the soil, crop, and human food chain [1], ultimately causing various human diseases [2]. In recent years, the use of rhizosphere microorganisms to regulate heavy metal accumulation in crops has become a research hotspot in the fields of pollution remediation and safe production. Differences in the composition of rhizosphere microbial communities are considered a key driver of the differentiation in heavy metal accumulation characteristics among plants varieties or ecotypes. Existing studies have reported associations between rhizosphere microbial diversity and Cd accumulation in different plant varieties. For instance, the low-Cd-accumulating rice variety XS14 was found to harbor higher rhizosphere microbial diversity than the high-Cd-accumulating variety YY17, and this higher diversity was associated with enhanced microecosystem stability and reduced Cd uptake [3]. Similarly, the low-Cd peanut variety FH1 exhibited higher Shannon, ACE, and Chao1 indices than the high-Cd variety SLH, and its Cd accumulation levels were negatively associated with these diversity indices [4]. Likewise, the low-Cd maize variety LD16 showed higher ACE, Shannon, and Observed Species indices than the high-Cd variety PY3 [5]. Taken together, these findings suggest that differences in rhizosphere bacterial diversity and composition may play a key role in regulating Cd accumulation in host plants. Systematic identification of functional strains from these communities holds promise for providing new microbial resources for the remediation of Cd-contaminated soils and the assurance of agricultural product safety.

Among the diverse microbial groups in the rhizosphere, bacteria were selected as the focus of this study for several considerations. Bacteria are known to colonize root surfaces at high densities and form biofilms that facilitate direct plant–microbe interactions [6,7,8]. Their rapid growth rates also allow for relatively quick physiological responses to environmental stimuli [9]. These characteristics make rhizosphere bacteria a tractable system for investigating microbial influences on plant Cd accumulation and offer valuable insights for the future design of potential microbiome-based intervention strategies. 

Rhizosphere bacteria modulate Cd uptake by plants through multiple mechanisms, which can be broadly categorized into physicochemical and biological pathways. The physicochemical pathway involves altering the rhizosphere chemical environment, such as raising or lowering soil pH, secreting extracellular polymers, organic acids, or siderophores, and thereby changing Cd bioavailability [10,11,12,13,14]. For example, *Burkholderia* sp. 1-22 and *Bacillus* sp. 6-6, isolated from rice rhizosphere, promote the conversion of exchangeable Cd to stable forms by raising soil pH and secreting extracellular polymers, thereby reducing Cd bioavailability and limiting plant uptake [10]. In contrast, *Ellin6067* and *Ramlibacter*, enriched in the rhizosphere of the high-Cd-accumulating wheat variety X128, lower rhizosphere pH, activate Cd, and promote Cd uptake [11]. *Enterobacter bugandensis* XY1 reduces acid-soluble heavy metals in soil through cell wall adsorption and chelation, decreasing Pb and Cd uptake by vegetables [12]. *Pseudomonas* sp. Lk9 increases Cd bioavailability by secreting organic acids and siderophores, promoting Cd accumulation in *Solanum nigrum* [13], while *Arthrobacter* sp. CC3 reduces Cd accumulation in soybean by raising soil pH and lowering Cd bioavailability [14]. The biological pathway involves direct interactions with plant roots, including the regulation of root development, cell wall remodeling, and modification of cell wall polysaccharide composition, which collectively influence Cd uptake and translocation [15,16,17,18]. For instance, *Sphingomonas* sp. SaMR12 promotes lateral root formation and enhances metal uptake in *Sedum alfredii* [15]. Similarly, inoculation with *Serratia* sp. IU01 and *Enterobacteriaceae* sp. IU02 stimulates lateral root proliferation in *Solanum nigrum*, expanding the root absorption surface and increasing Cd accumulation [16]. Likewise, strain Sy310 increases pectin and hemicellulose contents in *Salix matsudana* root cell walls under Cd stress, thereby enhancing Cd phytoextraction efficiency and plant tolerance [17]. In contrast, *Bacillus licheniformis* HDYM-04 and *Bacillus subtilis* ZC-01 secrete pectinase and mannanase to hydrolyze root cell wall polysaccharides in *Linum usitatissimum*, reducing pectin and hemicellulose content, decreasing Cd binding sites, and inhibiting Cd uptake [18]. Collectively, these findings indicate that rhizobacteria regulate Cd bioavailability and plant Cd accumulation through strain-specific pathways, involving both physicochemical modulation of the rhizosphere environment and direct biological interactions with root physiology and cell wall properties.

However, research on the regulatory effects of rhizosphere bacteria on Cd uptake in medicinal plants and the underlying mechanisms remains in its infancy, and systematic strain screening together with in depth mechanistic studies are still largely lacking. *Salvia miltiorrhiza* (Lamiaceae) is one of the most widely used medicinal species in clinical practice for promoting blood circulation and removing blood stasis [19], with its roots and rhizomes serving as the primary medicinal organs and also as the main target tissues for Cd accumulation. Previous studies have shown that Cd accumulation in *S. miltiorrhiza* differs significantly among ecotypes [20], and these variations are closely linked to root cell wall compositional traits, such as the degree of pectin demethylation and hemicellulose xylose content [21]. However, whether and how rhizosphere bacteria participate in modulating this ecotype-dependent Cd accumulation remain poorly understood. 

Against this backdrop, the present study employs two *S. miltiorrhiza* ecotypes, SC (which has low Cd accumulation) and SD (which has high Cd accumulation), as experimental materials to systematically compare the diversity and community composition of their rhizosphere bacterial assemblages, to identify key functional strains governing Cd accumulation, to characterize their regulatory effects on Cd uptake and partitioning in *S. miltiorrhiza*, and to elucidate the underlying mechanisms in depth. The findings provide specific strain resources and physiological theoretical bases for the future design of rhizobacteria-based intervention strategies aimed at integrating Cd reduction and quality improvement in medicinal plants in contaminated agricultural soils.

## 2. Results

### 2.1. Determination of an Appropriate Cd Concentration for Inducing Discernible Stress Responses in S. miltiorrhiza 

**Figure 1:**
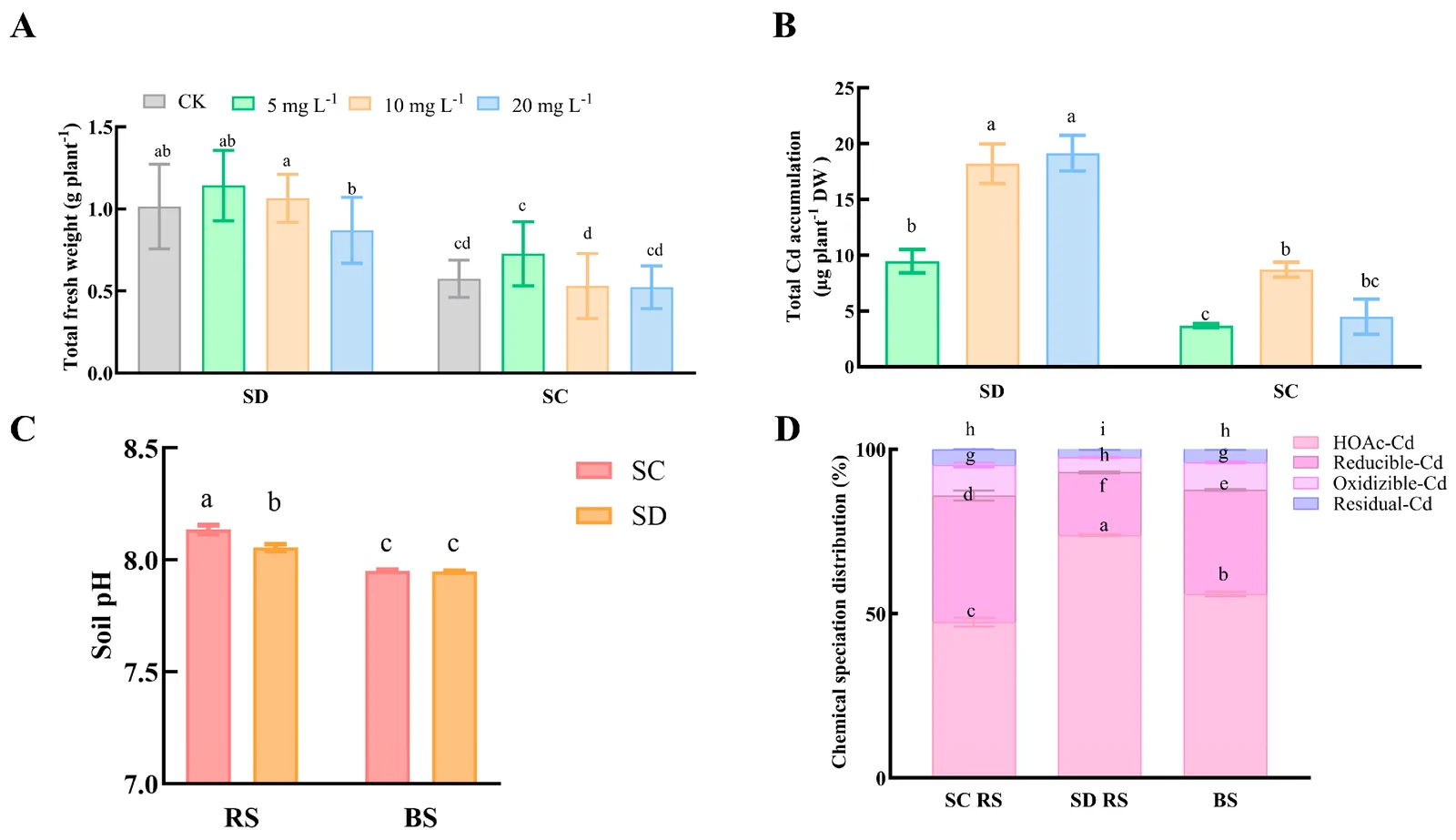
Effects of varying Cd concentrations on plant growth, Cd accumulation, and rhizosphere soil properties of high- or low-cd-accumulating ecotypes of *S. miltiorrhiza* (SD or SC). (**A**) Whole-plant fresh weight of SD and SC under different Cd concentrations (0, 5, 10, 20 mg·L^−1^). Data are presented as means ± SD (n = 6). Different lowercase letters indicate significant differences among groups, as determined by two-way analysis of variance (ANOVA) followed by Duncan’s multiple range test (*p* < 0.05). (**B**) Whole-plant Cd accumulation of SD and SC under different Cd concentrations (0, 5, 10, 20 mg·L^−1^). Data are presented as means ± SD (n = 3). Different lowercase letters indicate significant differences among Cd treatments and between ecotypes, as determined by two-way ANOVA followed by Tukey’s HSD post hoc test (*p* < 0.05). (**C**) Soil pH in the rhizosphere (RS) and bulk soil (BS) of SD and SC under 10 mg·kg^−1^ Cd treatment. Data are presented as means ± SD (n = 5). Different lowercase letters indicate significant differences between the two ecotypes within either the rhizosphere or the bulk soil, as determined by one-way ANOVA followed by Duncan’s multiple-range test (*p* < 0.05). (**D**) Chemical fractionation of Cd in the rhizosphere and bulk soil of SD and SC under 10 mg·kg^−1^ Cd treatment. Data are presented as means ± SD (n = 3). Data were analyzed using two-way ANOVA followed by Duncan’s multiple range test (*p* < 0.05). Different lowercase letters indicate significant differences among different soil samples and Cd chemical fraction treatments. SC: *S. miltiorrhiza* with low Cd accumulation; SD: *S. miltiorrhiza* with high Cd accumulation.

To determine the optimal Cd exposure level, SD and SC were grown in a nutrient soil, perlite, and vermiculite substrate with Cd additions at 0, 5, 10, and 20 mg·L^−1^. SD consistently exhibited a higher whole-plant fresh weight than SC across all treatments (Figure 1A). While 20 mg·L^−1^ Cd moderately inhibited SD growth (Figure 1A), Cd accumulation in SD did not differ significantly between 10 and 20 mg·L^−1^ treatments, whereas a slight decrease was observed in SC at 20 mg·L^−1^ (Figure 1B). Considering both growth performance and Cd accumulation, 10 mg·L^−1^ was chosen for subsequent assays, as it neither compromised plant growth nor obscured the ecotype-dependent difference in Cd accumulation, consistent with the classification of SD and SC as high- and low-Cd ecotypes [20].

### 2.2. Effects of SD and SC on the Rhizosphere Soil Microenvironment

To compare the rhizosphere effects of SD and SC, a pot experiment was performed in agricultural soil amended with 10 mg·kg^−1^ Cd. Bulk soil pH did not differ between the two ecotypes, whereas rhizosphere pH was significantly elevated relative to bulk soil in both, with SC showing significantly higher rhizosphere pH than SD (Figure 1C), indicating a marked divergence in rhizosphere microenvironment between the two ecotypes. Analysis of soil Cd chemical fractionation (Figure 1D) further revealed contrasting patterns between the two ecotypes. Compared with bulk soil, the proportion of HOAc-extractable Cd (representing the operationally defined exchangeable pool) was significantly increased in the rhizosphere of SD, but significantly decreased in that of SC. In contrast, the proportions of reducible and oxidizable Cd fractions were significantly reduced in the SD rhizosphere, whereas both fractions were markedly elevated in the SC rhizosphere. Concurrently, the residual Cd fraction (generally considered to be sparingly soluble and poorly mobile under normal soil conditions) also increased in the SC rhizosphere. Direct comparison between the two ecotypes within the rhizosphere further revealed that SC exhibited a significantly lower proportion of HOAc-extractable Cd and a significantly higher proportion of residual Cd than SD. Collectively, these results demonstrate that SD and SC differentially shape their rhizosphere microenvironments, leading to contrasting alterations in soil Cd chemical fractions, which were correlated with the differential Cd accumulation in *S. miltiorrhiza* roots.

### 2.3. Differences in Rhizosphere Bacterial Community Diversity and Composition Between SD and SC

Rhizosphere pH is closely linked to microbial community structure and metabolism, as bacteria can modulate pH and heavy metal speciation via proton and organic acid secretion [22]. To explore the rhizobacterial community differences between SD and SC and their link to Cd speciation, 16S rRNA high-throughput sequencing was performed.

Rarefaction analysis showed that ASV counts plateaued at 10,000~15,000 sequences, confirming adequate sequencing depth. SC consistently yielded higher ASV counts (2000~2300) than SD (1800~2100), indicating greater species richness in SC rhizosphere (Supplementary Figure S1A). Rank abundance curves showed a steep decline in dominant taxa with a long tail of rare species (Supplementary Figure S1B). Overall, the sequencing data are reliable and sufficient for subsequent analyses.

**Figure 2:**
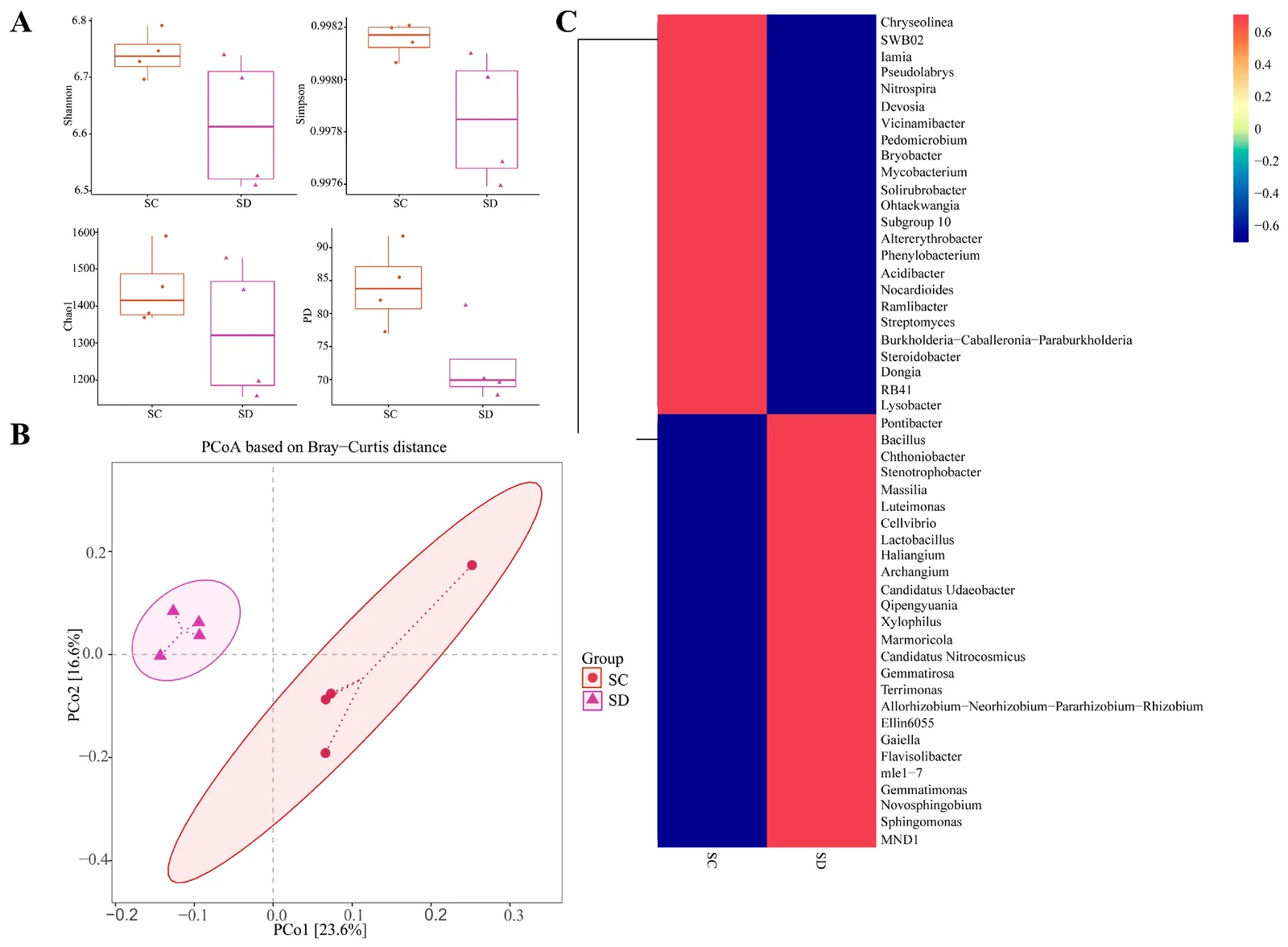
Rhizosphere bacterial diversity analysis of SD and SC. (**A**) *α*-diversity of rhizosphere bacteria in SD and SC; (**B**) *β*-diversity of rhizosphere bacteria in SD and SC based on PCoA analysis; (**C**) genus-level heatmap of rhizosphere bacteria in SD and SC. Note: SC refers to *S. miltiorrhiza* with low Cd accumulation, and SD refers to *S. miltiorrhiza* with high Cd accumulation. The Shannon index, Simpson index, and PD index of *α*-diversity are used to characterize intraspecific diversity; the Chao1 index is used to characterize intraspecific richness.

Analysis of *α*-diversity, based on the Shannon, Simpson, Chao1, and Faith Phylogenetic Diversity (PD) indices, revealed that SC rhizosphere bacterial communities harbored significantly higher species diversity and richness than those of SD (Figure 2A). Principal coordinate analysis (PCoA) based on Bray–Curtis distance revealed a clear separation between the two ecotypes (PCo1 = 23.6%, PCo2 = 16.6%), and this separation was statistically supported by PERMANOVA (Adonis, R^2^ = 0.25, *p* < 0.05), indicating a significant divergence in rhizosphere bacterial community structure between SC and SD (Figure 2B). At the genus level, 24 genera (e.g., *Chryseolinea*, *Nitrospira*) were significantly enriched in SC, whereas 26 genera (e.g., *Massilia*, *Sphingomonas*, and *Bacillus*) exhibited higher abundance in SD (Figure 2C). This differential bacterial distribution likely represents a key biological driver underlying the divergent Cd accumulation phenotypes between SD and SC. To further investigate whether these differentially abundant taxa directly modulate Cd accumulation in *S. miltiorrhiza*, we subsequently isolated and purified rhizosphere bacteria from both ecotypes and evaluated the effects of selected strains on Cd accumulation in the host plant.

### 2.4. Isolation and Cd Tolerance Evaluation of Cd-Tolerant Rhizobacteria from SD and SC

To isolate Cd-tolerant rhizobacteria, a dilution plating method was employed on NB agar medium supplemented with 10 mg·L^−1^ Cd. All single colonies that grew within 3~7 days were streak-purified and examined for colony morphology, color, and growth status. A total of 18 rhizobacterial strains were initially obtained from the rhizosphere soils of SD and SC (Supplementary Figure S2). Their Cd tolerance profiles were subsequently determined. 

**Figure 3:**
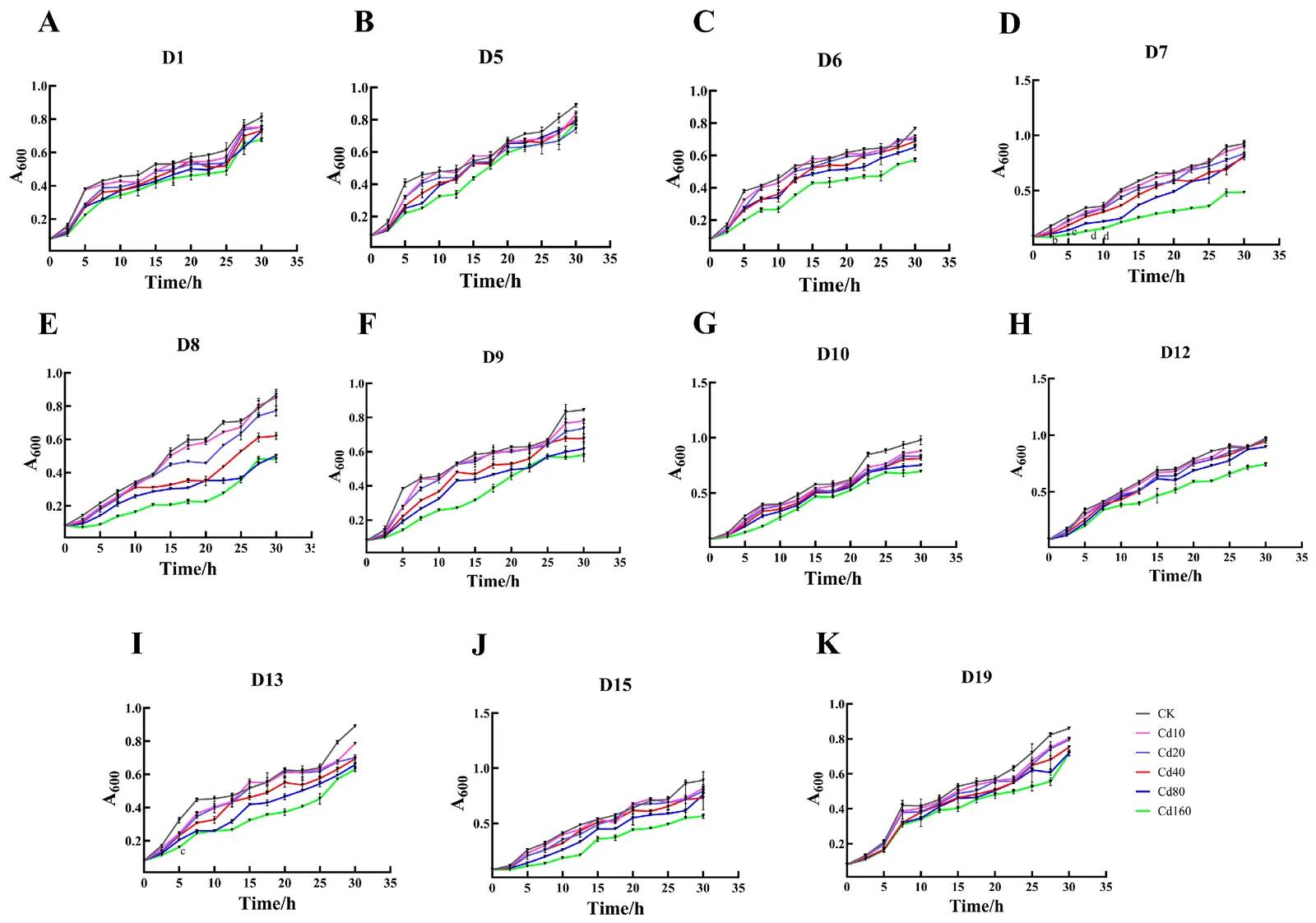
(**A**–**K**) Cd tolerance of rhizobacterial strains isolated from SD under Cd stress. Note: The initial A_600_ value for all strains was 0.08. CK denotes the blank control (no Cd treatment); Cd10, Cd20, Cd40, Cd80, and Cd160 represent Cd concentrations of 10, 20, 40, 80, and 160 mg·L^−1^, respectively.

Among them, 11 Cd-tolerant rhizobacterial strains were isolated from SD and purified on NB agar medium containing 10 mg·L^−1^ Cd (Figure 3A–K), with marked variations in Cd tolerance among the isolates. Strains D1, D5, D10, and D19 exhibited the highest Cd tolerance, showing only slight growth inhibition with increasing Cd concentrations (Figure 3A,B,G,K). Strains D6, D7, D9, D12, D13, and D15 also displayed relatively strong Cd tolerance, with moderate growth inhibition at 80 mg·L^−1^ Cd but pronounced inhibition at 160 mg·L^−1^ (Figure 3C,D,F,H–J). In contrast, strain D8 showed the weakest Cd tolerance, with growth significantly inhibited even at 40 mg·L^−1^ Cd (Figure 3E). Strain D8, with growth severely inhibited under Cd stress, was therefore excluded from further studies.

**Figure 4:**
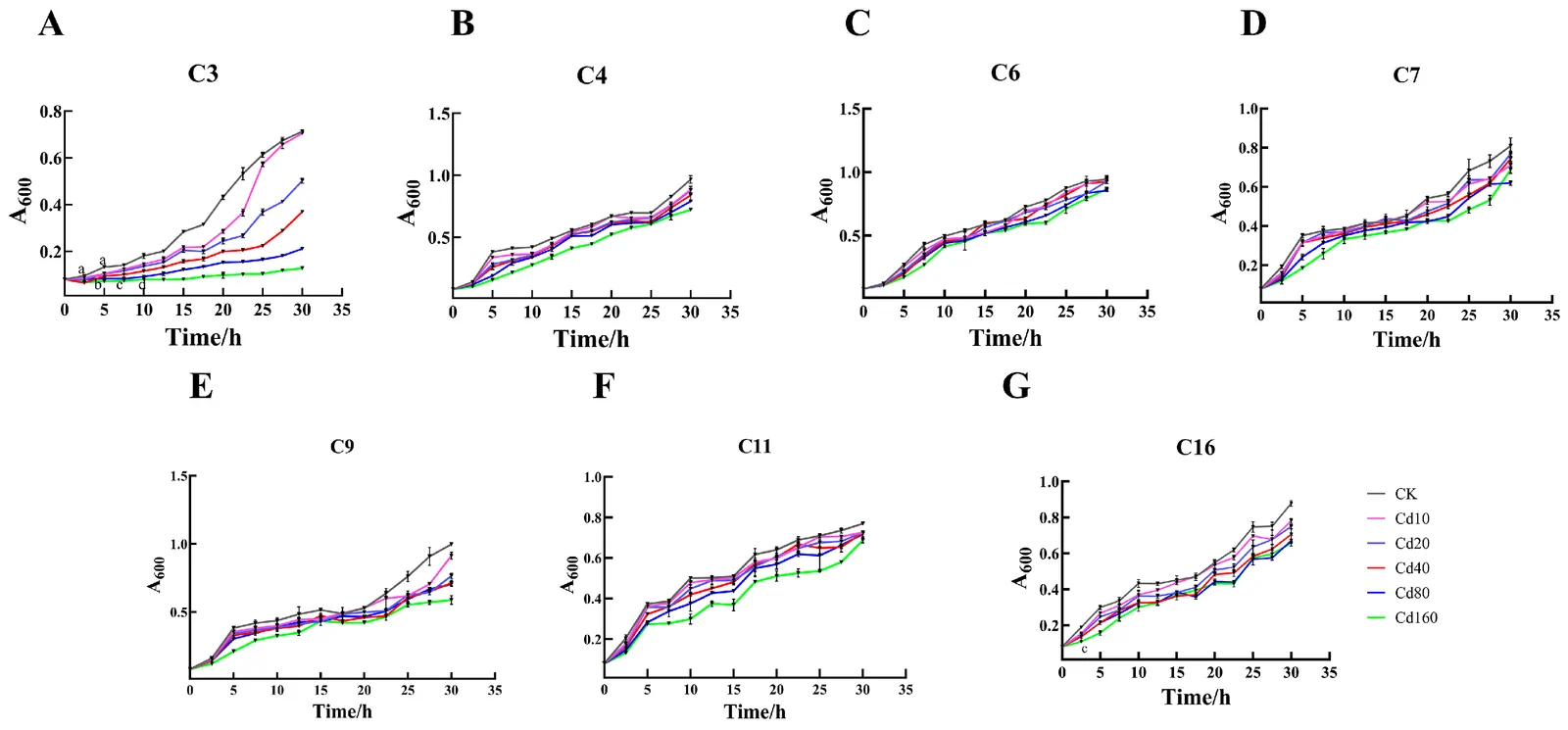
(**A**–**G**) Cd tolerance of rhizobacterial strains isolated from SC under Cd stress. Note: The initial A_600_ value for all strains was 0.08. CK denotes the blank control (no Cd treatment); Cd10, Cd20, Cd40, Cd80, and Cd160 represent Cd concentrations of 10, 20, 40, 80, and 160 mg·L^−1^, respectively.

Seven Cd-tolerant strains were isolated from SC and purified on NB agar medium containing 10 mg·L^−1^ Cd (Figure 4A–G), with marked variations in Cd tolerance among the isolates. Among these, strains C4, C6, C7, C11, and C16 exhibited relatively strong Cd tolerance, showing only slight growth inhibition under stepwise increasing Cd concentrations (Figure 4B–D,F,G), whereas strains C3 and C9 displayed weaker tolerance (Figure 4A,E). Strains C3 exhibited extremely low Cd tolerance, with growth severely inhibited under Cd stress, and was therefore excluded from further studies.

### 2.5. Effects of Cd-Tolerant Rhizobacterial Reinoculation on Growth and Cd Accumulation in S. miltiorrhiza

To screen for functional strains that significantly reduce or enhance Cd accumulation in *S. miltiorrhiza*, SD plants were cultivated in a nutrient substrate (soil:perlite:vermiculite = 3:0.5:0.5) amended with 10 mg·L^−1^ Cd. Sixteen Cd-tolerant strains (excluding the less tolerant D8 and C3) were individually reinoculated, and whole-plant fresh weight and Cd accumulation were subsequently determined (Table 1).

Under Cd stress, reinoculation with strains C7, C11, C16, D1, D5, D9, D15, and D19 significantly increased whole-plant fresh weight compared with the control, whereas strain C6 significantly suppressed plant growth. In terms of Cd accumulation, the 16 strains exhibited contrasting effects. Strains C4, C9, C16, D1, D10, D13, and D15 significantly reduced shoot Cd content, while C6, C7, D5, D6, D7, D9, D12, and D19 significantly increased it.

For root Cd content, C4, C16, and D13 exerted significant reductions, whereas C6, C7, C11, D1, D5, D6, D7, D9, D10, D12, D15, and D19 caused significant increases. In terms of Cd accumulation (content × biomass), C4 and C9 significantly decreased shoot Cd accumulation, while C6, C7, C11, C16, D1, D5, D6, D7, D9, D12, and D19 significantly increased it. Root Cd accumulation was significantly reduced by C4 and D13, but significantly enhanced by C6, C7, C11, C16, D1, D5, D6, D7, D9, D10, D12, D15, and D19. Consequently, whole-plant Cd accumulation was significantly decreased by C4 and D13, whereas it was significantly increased by C6, C7, C11, C16, D1, D5, D6, D7, D9, D10, D12, D15, and D19.

Collectively, strains C4 and D13 exhibited dual functionality in reducing Cd accumulation while promoting plant growth, whereas strains C7, C11, C16, D1, D5, D9, D15, and D19 showed the capacity to enhance both Cd accumulation and plant growth. Based on Cd tolerance assessment, C4, C7, C11, C16, D9, D13, and D15 were identified as superior candidates. Using root (the medicinal organ) Cd accumulation and whole-plant Cd accumulation as core screening criteria, two strains, C4 and D9, were ultimately selected as key functional strains. Strain C4 significantly reduced Cd accumulation in shoots, roots, and whole plants while promoting plant growth, making it the most effective strain for reducing Cd uptake in *S. miltiorrhiza*. In contrast, strain D9 significantly enhanced Cd accumulation across all plant compartments and the whole plant while also promoting growth, positioning it as the optimal strain for improving Cd tolerance and phytoextraction capacity. The regulatory mechanisms of these two representative strains were further investigated through subsequent inoculation experiments.

### 2.6. Molecular Identification and Cd Adsorption Kinetics of Functional Strains C4 and D9

**Figure 5:**
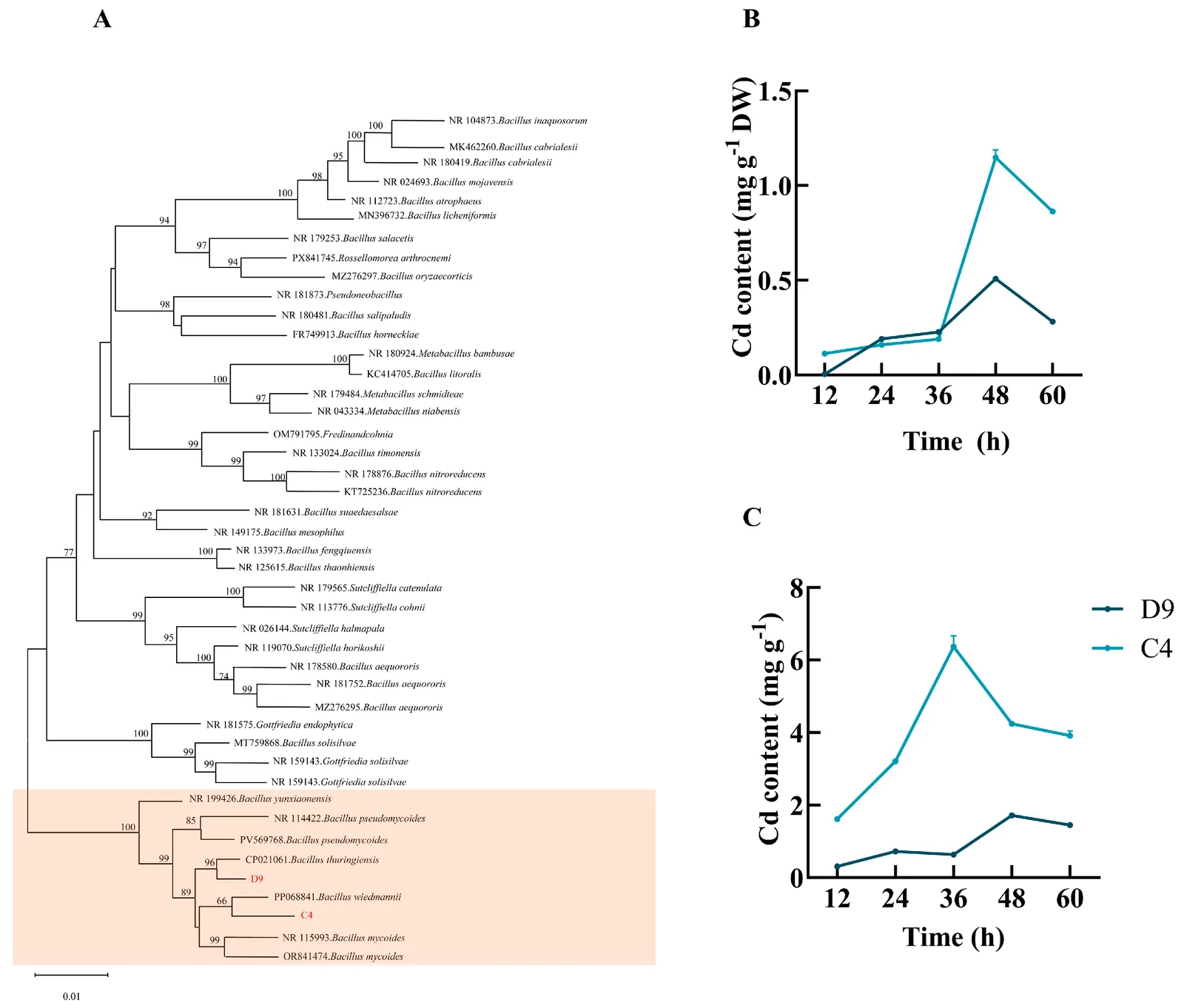
Molecular characterization of functional strains C4 and D9 and their Cd^2+^ adsorption kinetics. (**A**) Phylogenetic tree of strains C4 and D9 based on 16S rRNA gene sequences; (**B**) intracellular Cd^2+^ accumulation in strains C4 and D9 over 60 h (n = 3); (**C**) extracellular Cd^2+^ binding in strains C4 and D9 over 60 h (n = 3).

To determine the taxonomic affiliation of strains C4 and D9, the 16S rRNA gene amplicons were verified by gel electrophoresis and subjected to paired-end sequencing (Chengdu Qingke Biotechnology Co., Ltd., Chengdu, China). The resulting sequences were compared against the NCBI database (https://www.ncbi.nlm.nih.gov/) using BLAST in NCBI, and a phylogenetic tree was constructed with MEGA 12.1 (Figure 5A). Both C4 and D9 were assigned to the genus *Bacillus* and clustered within the *Bacillus cereus* group. Strain C4 formed a clade with the representative strain of *B. wiedmannii*, albeit with low bootstrap support (66%), indicating that 16S rRNA gene sequences alone are insufficient for species-level resolution of C4 from closely related taxa. Strain D9 clustered with *B. thuringiensis* with high bootstrap support (96%), indicating a close phylogenetic relationship. However, despite this close relatedness, strain D9 was preliminarily identified as a member of the *B. cereus* group within the genus *Bacillus*, rather than as *B. thuringiensis* per se. Therefore, multilocus sequence typing (MLST) based on housekeeping genes such as *gyrB* and *rpoB*, or whole-genome sequencing with average nucleotide identity (ANI) analysis, will be required for accurate species delineation of strain C4 and D9.

The Cd adsorption kinetics of strains C4 and D9 exhibited distinct temporal dynamics. Intracellular Cd accumulation increased progressively with incubation time, peaking at 48 h for both strains (C4: 1.15 mg·g^−1^; D9: 0.51 mg·g^−1^), followed by a gradual decline (Figure 5B). Extracellular Cd binding reached its maximum at 36 h for C4 (6.37 mg·g^−1^) and at 48 h for D9 (1.71 mg·g^−1^), also decreasing subsequently (Figure 5C). These results suggest that one mechanism underlying the reduced Cd uptake in plants inoculated with C4 may involve the sequestration of Cd by bacterial cells, including both intracellular accumulation and extracellular binding, thereby potentially reducing Cd bioavailability in the rhizosphere.

### 2.7. Effects of Inoculation with Strains C4 and D9 on Rhizosphere Soil Microenvironment and Cd Chemical Fractionation in SD

**Figure 6:**
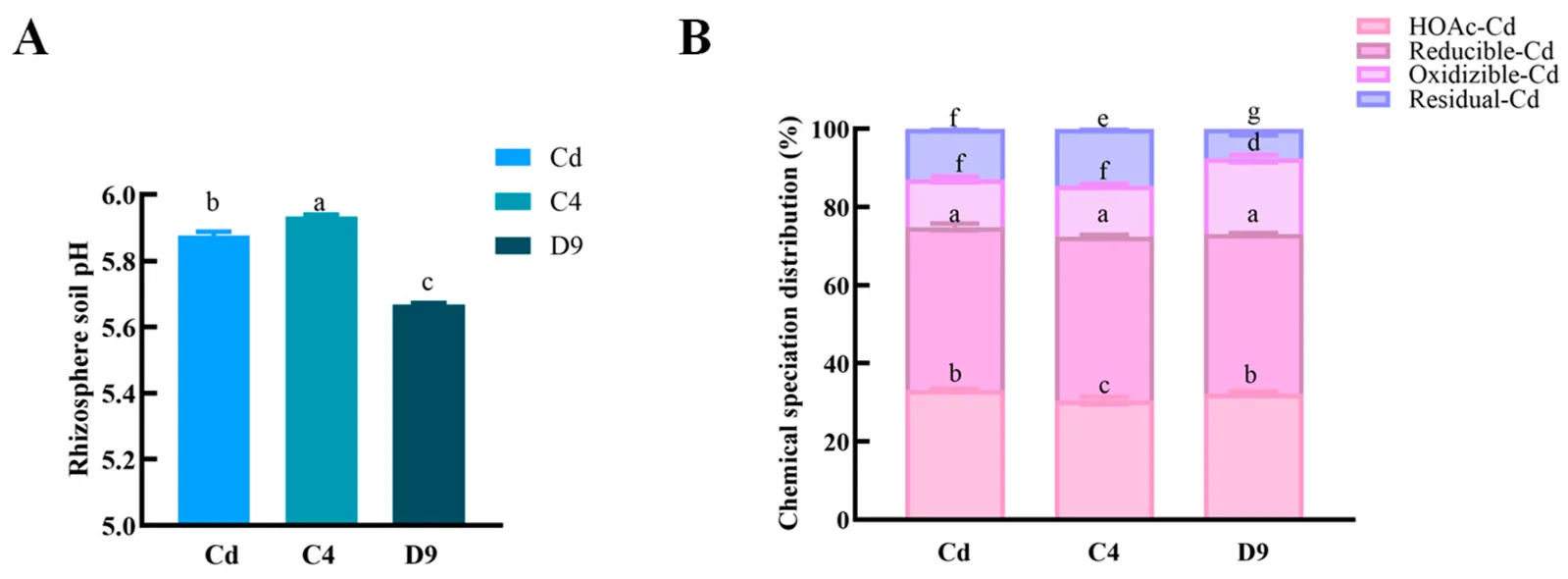
Effects of inoculation with functional strains D9 and C4 on rhizosphere soil pH and Cd chemical fractionation in *S. miltiorrhiza*. (**A**) Rhizosphere soil pH. Values are means ± SD (n = 3). Different lowercase letters denote significant differences among treatments at *p* < 0.05, as determined by one-way ANOVA followed by Duncan’s multiple-range test. (**B**) Proportions of different Cd chemical fractions in rhizosphere soil under different treatments. Values are means ± SD (n = 3). Different lowercase letters indicate significant differences at *p* < 0.05, as assessed by one-way ANOVA with Duncan’s multiple-range test. Cd, 10 mg·L^−1^ Cd stress (non-inoculated control); C4, 10 mg·L^−1^ Cd stress + *Bacillus* sp. C4; D9, 10 mg·L^−1^ Cd stress + *Bacillus* sp. D9.

To investigate the effects of strains C4 and D9 on rhizosphere soil properties, plant performance, and root cell wall chemistry in *S. miltiorrhiza*, SD plants were cultivated in a nutrient substrate (soil:perlite:vermiculite = 3:0.5:0.5, v/v/v) amended with 10 mg·L^−1^ Cd and inoculated with C4 or D9, respectively. The results showed that bacterial inoculation significantly altered rhizosphere soil pH and Cd chemical fractionation. Inoculation with C4 increased rhizosphere pH by 0.05 units, whereas D9 decreased it by 0.26 units (Figure 6A). Moreover, the two strains exerted contrasting effects on Cd chemical fractionation (Figure 6B). Compared with the control, C4 inoculation significantly reduced the proportion of HOAc-extractable Cd fraction (the most labile and readily mobilized pool) by 2.62% and increased the residual Cd fraction by 1.61%. In contrast, D9 inoculation significantly increased the oxidizable Cd fraction by 7.12% and decreased the residual Cd fraction by 5.36% (Figure 6B).

### 2.8. Effects of Inoculation with Strains C4 and D9 on Growth and Root Morphology of S. miltiorrhiza

Compared with the treatment containing Cd only, inoculation with strain C4 significantly increased the shoot height, total plant height, shoot fresh weight, and average root diameter of SD plants (Supplementary Figure S3B,C,E,H), whereas strain D9 significantly increased root fresh weight and average root diameter (Supplementary Figure S3D,H). Although the increases in root fresh weight and whole-plant fresh weight did not reach statistical significance (*p* > 0.05), C4 inoculation increased root fresh weight by 20.19% and whole-plant fresh weight by 21.35% (Supplementary Figure S3D,F), while D9 inoculation increased these parameters by 34.62% and 18.42%, respectively (Supplementary Figure S3D,F). Collectively, these results indicate that D9 significantly promotes the root fresh weight of *S. miltiorrhiza*, while C4 showed a numerical increase that did not reach statistical significance. Furthermore, neither strain significantly affected root length, but both significantly increased root diameter (Supplementary Figure S3H), suggesting that they reshape root morphological architecture.

### 2.9. Effects of Inoculation with Strains C4 and D9 on Subcellular Cd Distribution and Root Cell Wall Polysaccharide Composition in S. miltiorrhiza

**Figure 7:**
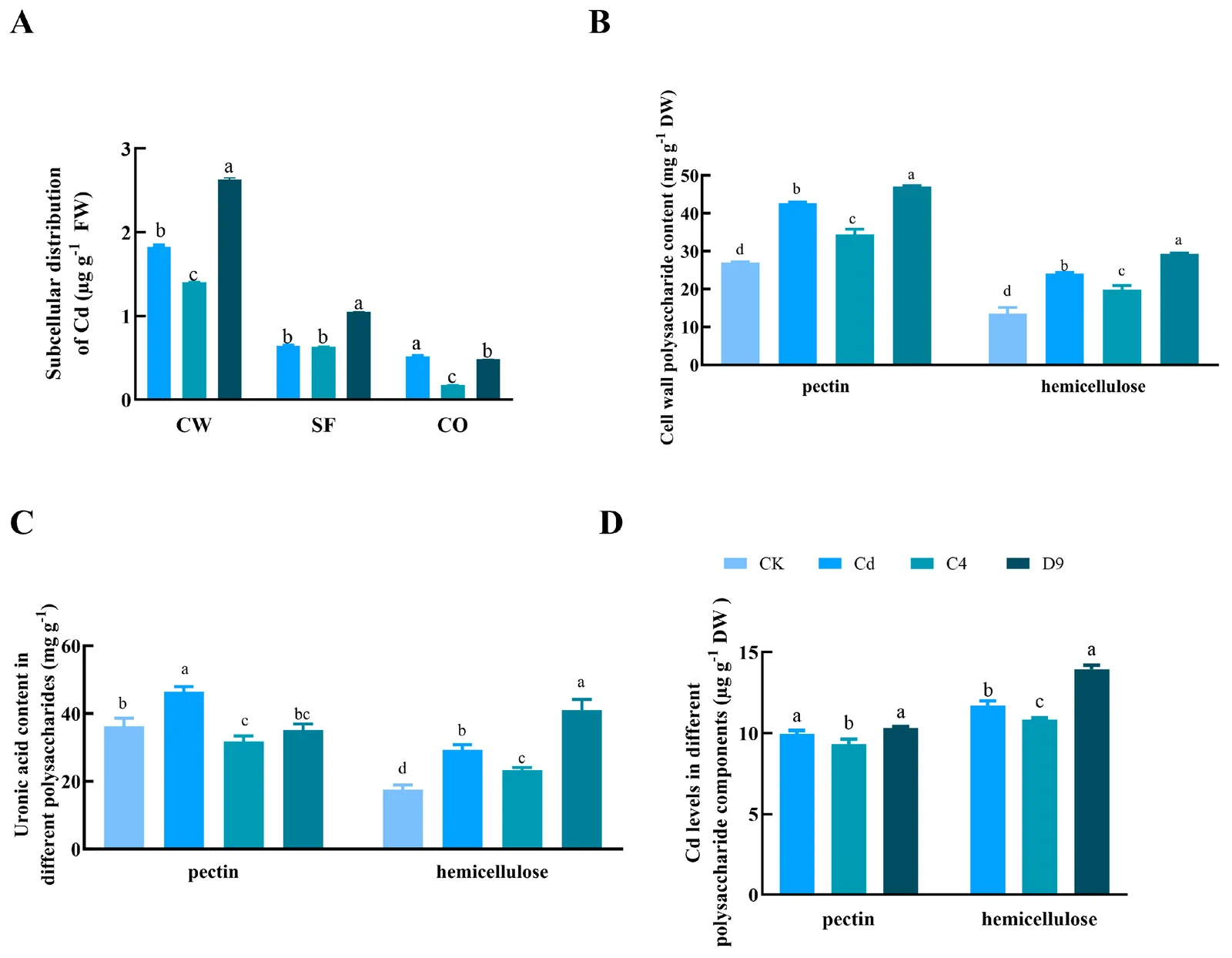
Subcellular Cd distribution and cell wall polysaccharide characteristics in roots of *S. miltiorrhiza* under Cd stress. (**A**) Cd content in root subcellular fractions. Data are presented as means ± SD (n = 3). CW, cell wall; SF, soluble fraction; CO, organelles. (**B**) Pectin and hemicellulose contents in root cell walls. (**C**) Uronic acid contents in pectin and hemicellulose fractions of root cell walls. (**D**) Cd content associated with pectin and hemicellulose fractions of root cell walls. Data are presented as means ± SD (n = 3). Different letters indicate significant differences in the same polysaccharide fraction among treatments, as determined by one-way ANOVA followed by Duncan’s multiple-range test (*p* < 0.05). CK, 0 mg·L^−1^ Cd without bacterial inoculation; Cd, 10 mg·L^−1^ Cd without bacterial inoculation; C4, 10 mg·L^−1^ Cd + *Bacillus* sp. C4; D9, 10 mg·L^−1^ Cd + *Bacillus* sp. D9.

To elucidate the Cd enrichment mechanisms in *S. miltiorrhiza* roots under Cd stress, the subcellular distribution of Cd (cell wall, soluble fraction, and organelles) in SD roots was analyzed. Under all treatments, Cd was predominantly localized in the cell wall, followed by the soluble fraction, with the lowest proportion in organelles. Inoculation with C4 significantly reduced Cd content in both the cell wall and organelles, whereas D9 inoculation significantly increased Cd content in the cell wall and soluble fraction, suggesting that the cell wall serves as a critical site for Cd immobilization in roots (Figure 7A). 

Further analysis of root cell wall polysaccharide content revealed that C4 inoculation significantly decreased the contents of pectin and hemicellulose, as well as the uronic acid content in these polysaccharides. In contrast, D9 inoculation significantly increased the contents of pectin and hemicellulose, along with the uronic acid content in hemicellulose (Figure 7B,C). Given the close association between hemicellulose/pectin content and the Cd binding capacity of the cell wall, we further determined the Cd content associated with each polysaccharide fraction. C4 inoculation significantly reduced Cd content in both pectin and hemicellulose fractions, whereas D9 inoculation significantly increased Cd content in the hemicellulose fraction (Figure 7D). Collectively, these results indicate that the two functional strains may modulate Cd adsorption and chemical complexation in *S. miltiorrhiza* by remodeling root cell wall polysaccharide composition.

### 2.10. Effects of Inoculation with Strains C4 and D9 on the Accumulation of Pharmacopeia-Indicated Bioactive Compounds in S. miltiorrhiza

**Figure 8:**
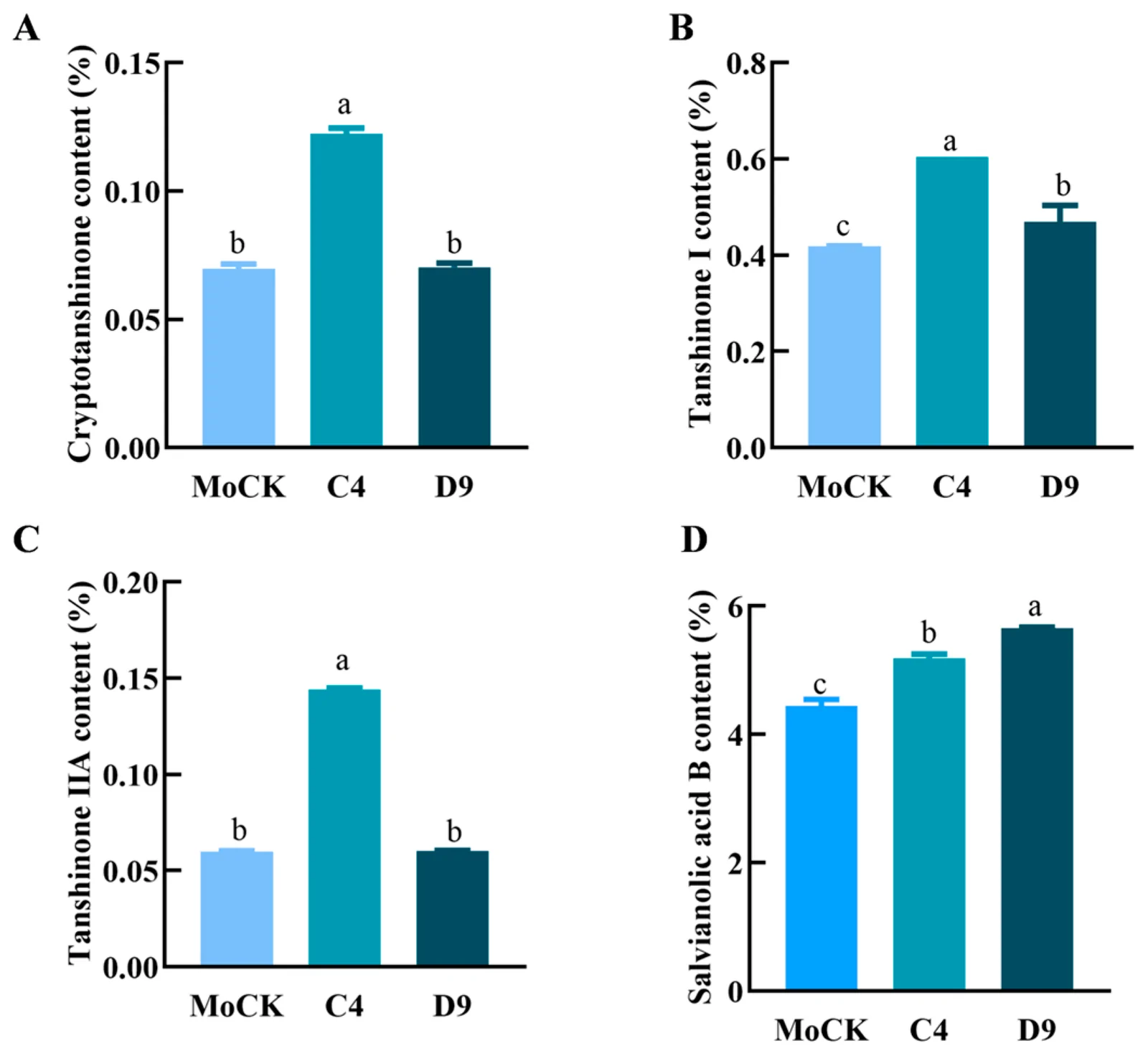
Effects of inoculation with strains C4 and D9 on the contents of four pharmacopeia-indicated bioactive compounds in *S. miltiorrhiza* under Cd stress. (**A**) Cryptotanshinone content. (**B**) Tanshinone I content. (**C**) Tanshinone IIA content. (**D**) Salvianolic acid B content. MoCK, soil Cd content 1.3 mg·kg^−1^; C4, soil Cd content 1.3 mg·kg^−1^ + *Bacillus* sp. C4; D9, soil Cd content 1.3 mg·kg^−1^ + *Bacillus* sp. D9. Data are presented as means ± SD (n = 3). Different letters indicate significant differences among treatments, as determined by one-way ANOVA followed by Duncan’s multiple-range test (*p* < 0.05).

To evaluate the potential of these two functional strains in practical application, we used natural Cd-contaminated soil (1.3 mg·kg^−1^) to simulate field conditions, inoculated C4 and D9 strains into SD separately, and detected their effects on the pharmacopeia constituents. Inoculation with C4 significantly increased the contents of tanshinones, with cryptotanshinone, tanshinone I, and tanshinone IIA elevated by 74.96%, 44.33%, and 140.33%, respectively, along with a 16.61% increase in salvianolic acid B (Figure 8). D9 inoculation resulted in more modest increases, with only tanshinone I and salvianolic acid B showing significant elevations of 12.11% and 27.19%, respectively (Figure 8B,D). These results indicate that C4 inoculation is associated with substantially greater accumulation of both lipophilic tanshinones and phenolic acids compared with D9, suggesting a more pronounced effect of C4 on secondary metabolite accumulation in *S. miltiorrhiza*. However, since the physicochemical properties of the naturally Cd-contaminated soil (particularly pH and organic matter) were not determined in the present study, the lack of these data may limit the interpretation of Cd availability, suggesting that future studies should include detailed soil physicochemical analyses to strengthen such interpretations.

## 3. Discussion

### 3.1. Two Bacillus Strains Differentially Regulate Cd Accumulation in S. miltiorrhiza Roots

Inoculation with *Bacillus* sp. C4 suppressed Cd accumulation in *S. miltiorrhiza* roots, whereas *Bacillus* sp. D9 exerted the opposite effect, promoting Cd accumulation. Despite sharing the same genus, the two strains exhibited markedly divergent regulatory effects on root Cd accumulation. Functional divergence among congeneric rhizobacterial strains is well documented. For instance, *Pseudomonas aeruginosa* KUCd1 promotes Cd accumulation in *Cucurbita moschata* [23], while *Pseudomonas* sp. W112 significantly reduces Cd accumulation in wheat [24]. Similar functional differentiation has also been reported within the genus *Bacillus*. *B. siamensis* R27 decreases Cd accumulation in lettuce seedlings [25], whereas *B. licheniformis* promotes Cd uptake in *Solanum nigrum* while alleviating Cd toxicity [26].

Mechanistic studies have demonstrated that *Rhizobium pusense* KG2 effectively prevents Cd uptake in soybean roots through cell wall immobilization of Cd and modification of rhizosphere soil structure [27]. Similarly, *Pseudomonas aeruginosa* immobilizes soil Cd via adsorption and precipitation on the bacterial cell wall, thereby reducing Cd content in rice grains [28]. Our Cd adsorption kinetic analysis revealed that *Bacillus* sp. D9 exhibited significantly lower Cd accumulation capacity than *Bacillus* sp. C4. These findings suggest that inoculation with *Bacillus* sp. C4 was associated with reduced Cd accumulation in *S. miltiorrhiza* and a concurrent decrease in the labile Cd fraction in the rhizosphere, suggesting a potential link between Cd fraction shifts and plant Cd uptake. 

Importantly, the genus-level heatmap of rhizosphere bacteria in SD and SC (Figure 2C) showed that the genera *Bacillus* was significantly more abundant in the SD rhizosphere than in the SC rhizosphere. While these genus-level abundance patterns provide ecological context, they do not directly explain the isolation origins or functional characteristics of C4 and D9, particularly given that *Bacillus* was more abundant in SD, yet C4 (a *Bacillus* strain) originated from SC. Therefore, we interpret the rhizosphere community analysis and the cultivation-based functional screening as complementary lines of evidence that together offer a broader view of the ecotype-associated microbiome, rather than implying a direct causal link between community composition and the specific functions of the isolated strains.

### 3.2. Rhizobacterial Regulation of Soil Cd Fractions Is Associated with Shifts in Rhizosphere pH

Cd-tolerant bacteria have been shown to be effective agents for bioremediation of Cd-contaminated soils, promoting plant growth through multiple pathways [29]. These microorganisms can absorb, transform, and immobilize soil Cd through their metabolic activities, thereby reducing its bioavailability [30]. They can also indirectly influence Cd mobility by modulating soil physicochemical properties, particularly pH, which is a key factor governing Cd partitioning in soil, with soil pH being negatively correlated with Cd bioavailability. Under Cd stress, *Rhizobium* sp. WW22, *Burkholderia* sp. WW13, and *Pseudomonas* sp. WW2 have been reported to secrete organic acid anions (e.g., itaconate), which act as alkaline equivalents by consuming H^+^ or displacing OH^−^ adsorbed on soil colloids, thereby exerting a net pH elevating effect. Within 10 days, these strains increased soil pH by 0.01~0.32 units and reduced exchangeable Cd content by 2.22~9.19%. Furthermore, the organic acid anions competitively displaced Cd^2+^ from soil adsorption sites, promoting the transformation of Cd from exchangeable to more stable forms, with exchangeable Cd further decreasing by 4.09~5.86% after 14 days [31]. Similarly, *Arthrobacter* sp. CC3, *Pseudarthrobacter* sp. CC12, and *Mesorhizobium* sp. CC13 have been shown to reduce soil Cd bioavailability and decrease Cd accumulation in soybean [14]. In contrast, inoculation with *E. coli* 10527 decreased soil pH, mobilized sparingly soluble Cd, and upregulated rhizosphere metabolic pathways, leading to increased amino acid and organic acid concentrations, which in turn promoted Cd uptake and translocation in *Suaeda salsa*, resulting in a 24.51~73.21% increase in total Cd accumulation [32]. Likewise, inoculation with *Bacillus subtilis* in the rhizosphere of *Sedum alfredii* significantly increased shoot accumulation of Cd, Zn, Cu, and Pb, an effect closely linked to strain induced rhizosphere acidification and enhanced metal bioavailability [33].

In the present study, inoculation with *Bacillus* sp. C4 significantly elevated soil pH and reduced the HOAc-extractable Cd fraction, correlating with reduced plant Cd uptake and accumulation. Conversely, inoculation with *Bacillus* sp. D9 decreased soil pH and increased the HOAc-extractable Cd fraction, corresponding with enhanced Cd accumulation in plants. These findings suggest a possible mechanistic link between pH modulation and Cd fractionation. One plausible explanation is that C4 may elevate rhizosphere pH through metabolites such as organic acid anions, potentially driving Cd transformation from the HOAc-extractable fraction to more stable forms (e.g., residual fraction). Conversely, D9 may mobilize sparingly soluble Cd through rhizosphere acidification, thereby increasing the labile Cd pool. However, these proposed mechanisms were not directly tested in this study and should be interpreted as hypothetical working models to guide future investigation rather than as established processes.

### 3.3. Differential Responses of Pectin and Hemicellulose to Rhizobacterial Inoculation

The plant root cell wall serves as the primary physical and chemical barrier against Cd uptake, primarily through the coordination and immobilization of Cd by functional groups in polysaccharide components such as pectin, hemicellulose and cellulose [21,34,35]. Among these, pectin and hemicellulose are responsible for immobilizing over 80% of root-associated Cd, playing a pivotal role in metal binding within the cell wall [36].

In the present study, inoculation with *Bacillus* sp. D9 increased hemicellulose content in the root cell walls of *S. miltiorrhiza*, thereby enhancing Cd sequestration capacity. This observation is consistent with findings in *Salix matsudana*, where strain Sy310 increased hemicellulose content and pectin methylesterase (PME) activity, leading to increased abundance of carboxyl (-COOH) and hydroxyl (-OH) groups and consequently enhanced Cd immobilization [17]. Similarly, rhizobacteria have been reported to enhance rice tolerance to aluminum by modifying hemicellulose composition [37], and *Azospirillum brasilense* promotes cell wall synthesis and inhibits heavy metal translocation to shoots through upregulation of cell wall synthase-related genes [38]. Collectively, these lines of evidence suggest that *Bacillus* sp. D9 may operate through a comparable mechanism, promoting hemicellulose synthesis and increasing Cd partitioning to the cell wall, thereby compartmentalizing Cd within the wall and enhancing Cd tolerance in *S. miltiorrhiza*.

In contrast, inoculation with *Bacillus* sp. C4 resulted in decreased pectin and hemicellulose contents in *S. miltiorrhiza* roots, reducing Cd binding sites in the cell wall and thereby attenuating root Cd immobilization. Similar changes in cell wall polysaccharide contents have been observed in other plant–microbe systems. For instance, *Azospirillum* strains have been reported to induce increased activity of pectin degrading enzymes, leading to reduced pectin content [39]; *Tsukamurella tyrosinosolvens* P9 and *Bacillus amyloliquefaciens* Bs006 have been associated with upregulation of pectin esterase genes and enhanced pectin degradation in peanut and banana, respectively [40,41]; and *Weissella paramesenteroides* and *Paenibacillus* sp. have been shown to correlate negatively with hemicellulose content, suggesting potential hemicellulose degradation [42]. These reports support the plausibility that *Bacillus* sp. C4 may influence root cell wall polysaccharide contents. However, the specific mechanisms underlying the observed reduction in pectin and hemicellulose—whether through bacterial secreted enzymes, plant gene regulation, or other processes—were not directly investigated in this study and remain to be elucidated.

Importantly, the present study did not assess the root colonization behavior of strains C4 and D9. It therefore remains ambiguous whether the observed phenotypic effects originate from sustained in situ rhizosphere activities of the introduced strains or from a transient elicitation effect driven by extracellular metabolites produced during the initial inoculation. Differentiating between these two scenarios is essential for elucidating the functional mode of action and for rational design of inoculation protocols. Moreover, although C4 and D9 are native isolates from two distinct cultivated ecotypes of *S. miltiorrhiza* (SC and SD), these ecotypes differ in multiple agronomic traits. Consequently, prior to any agricultural or translational deployment, comprehensive genomic profiling—particularly screening for toxin biosynthesis- and virulence-associated gene clusters—is warranted to ensure strain safety and regulatory compliance. Collectively, these findings provide microbial resources for the future development of biofertilizers aimed at modulating the quality of *S. miltiorrhiza* cultivated in Cd-contaminated soils.

**Figure 9:**
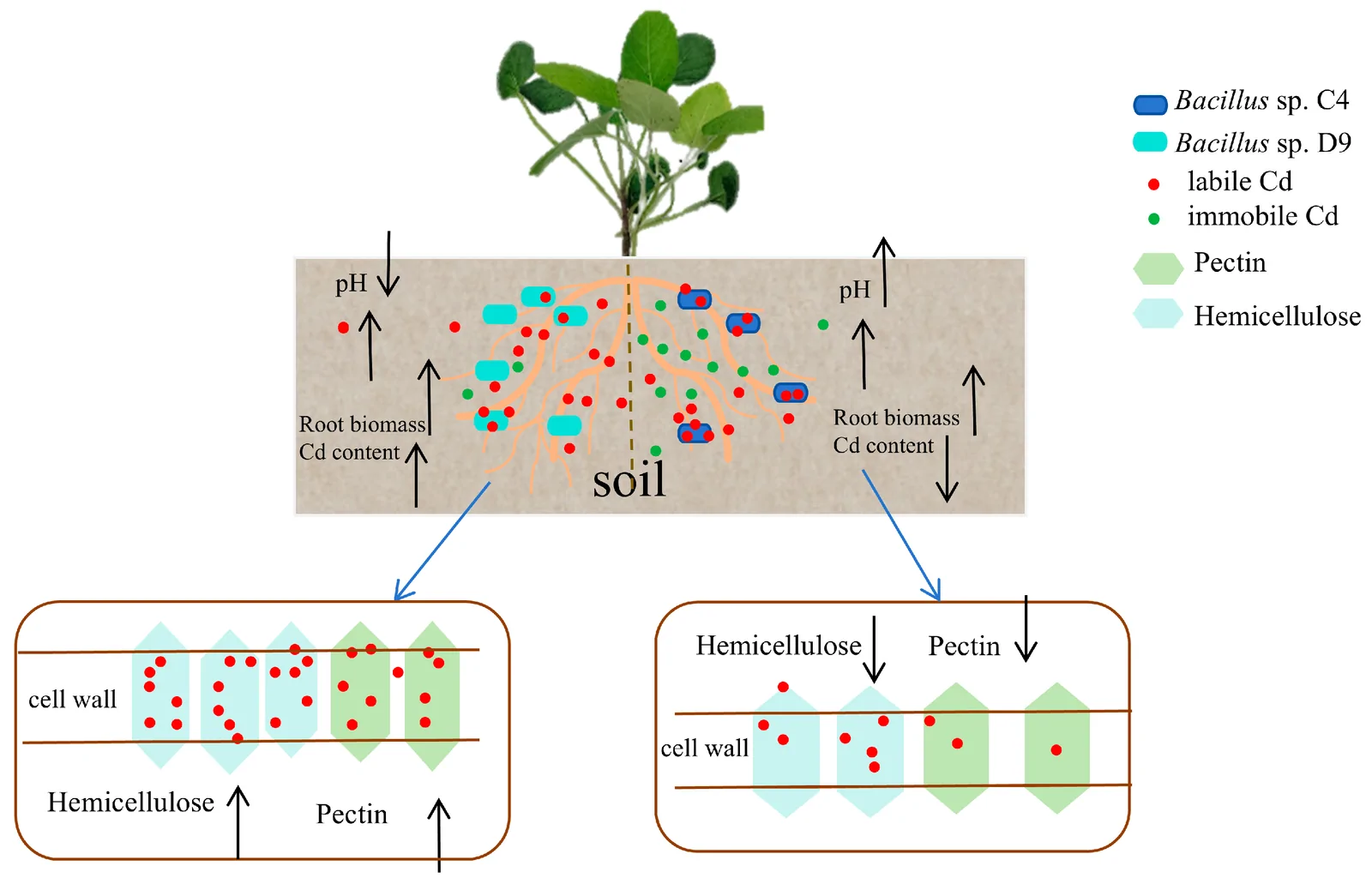
Proposed mechanistic model depicting the contrasting regulatory pathways of *Bacillus* sp. C4 and D9 on Cd accumulation in *S. miltiorrhiza*.

Taken together, our findings demonstrate that strain C4 reduces Cd accumulation in *S. miltiorrhiza*, which was associated with rhizosphere alkalinization, reduced pectin and hemicellulose content in root cell walls, and Cd accumulation by bacterial cells, corresponding with restricted Cd influx, enhanced plant growth, and increased active constituent accumulation. In contrast, strain D9 promotes Cd uptake and biomass production, which was associated with rhizosphere acidification, hemicellulose enrichment in root cell walls, and altered root morphology. To provide an integrated overview, we propose a working model summarizing the key regulatory network (Figure 9).

## 4. Materials and Methods

### 4.1. Plant Materials

The *S. miltiorrhiza* ecotypes used in this study, namely SD (which has high Cd accumulation) and SC (which has low Cd accumulation), were previously identified and screened by our research group [20]. Plantlets of both ecotypes were obtained via tissue culture following the method described by Yao et al. [43].

### 4.2. Screening of Cd Treatment Concentration

To determine the appropriate Cd concentration for subsequent experiments, SD and SC were subjected to four Cd treatment levels: 0 (control), 5, 10, and 20 mg·L^−1^. Cd was supplied as CdCl_2_·2.5H_2_O. The cultivation substrate was composed of nutrient soil, vermiculite, and perlite at a ratio of 3:0.5:0.5 (v/v/v). Each treatment was replicated three times. Plants were grown in 8 cm × 8 cm plastic pots, with one seedling per pot and ten pots per treatment. Immediately after transplantation, Cd treatment was initiated by root irrigation and repeated every three days. Plant samples were collected for analysis after 15 days of treatment.

### 4.3. Pot Experiment Setup

The test soil was collected from the farm of the Ya’an Campus of Sichuan Agricultural University, representing agricultural soil with no prior history of *S. miltiorrhiza* cultivation. A Cd solution of 10 mg·kg^−1^, prepared using CdCl_2_·2.5H_2_O, was added to the test soil, thoroughly mixed, and then equilibrated for 2 months to ensure homogeneous Cd distribution, according to established protocols [44]. Concurrently, in vitro propagated plantlets of SD and SC were maintained for 60 days and then transplanted into the equilibrated soil, where they were grown for an additional 30 days [45]. Plants were grown in plastic pots (42 cm × 40 cm) containing 8 kg of soil per pot, with five plantlets per pot and three replicate pots per treatment. 

### 4.4. Plant Phenotypic and Root Morphological Analysis

For plant phenotypic measurement, plants were carefully washed, blotted dry, and then assessed for shoot height, root length, and fresh weight, with 5~6 plants measured per treatment. For root morphological observation, soil particles adhering to the root surface were gently removed with deionized water, with great care taken to preserve the integrity of the root system. The roots were then fully spread in water and scanned using a root scanner (ScanMaker i800 Plus, Shanghai Microtek Technology Co., Ltd., Shanghai, China) [46]. The root surface area, total root length, average root diameter, and number of root tips were analyzed, with 5 plants scanned per treatment.

### 4.5. Soil pH Determination

After 30 days of cultivation, rhizosphere and bulk soils were separately collected from SD and SC treatments. Soil samples were passed through a 2 mm sieve to remove stones and root debris, placed into sample bags, and thoroughly mixed. For pH measurement, 10.0 g of each soil sample was weighed and mixed with 50 mL of deionized water, stirred thoroughly, and allowed to stand for 1 h. The supernatant was then used for pH determination [47]. Each treatment included three replicates.

### 4.6. Sequential Extraction of Soil Cd Fractions

Chemical speciation of Cd in soil was determined using the sequential extraction procedure proposed by the European Community Bureau of Reference (BCR). Soil samples that were air-dried and passed through a 2 mm sieve (1.0 g) were subjected to a four-step extraction to determine the acid-extractable Cd (HOAc-Cd), reducible Cd (Reducible-Cd), oxidizable Cd (Oxidizable-Cd), and residual Cd (Residual-Cd) fractions.

Step 1 (acid-soluble fraction): A total of 40 mL of 0.11 mol·L^−1^ CH_3_COOH was added, and the mixture was shaken at room temperature for 16 h. The supernatant after centrifugation was collected for HOAc-Cd determination.

Step 2 (reducible fraction): The residue from Step 1 was extracted with 40 mL of 0.5 mol·L^−1^ NH_3_OH·HCl and 0.05 mol·L^−1^ HNO_3_ and shaken at room temperature for 16 h, and the supernatant was collected.

Step 3 (oxidizable fraction): The residue from Step 2 was digested twice with 10 mL of 30% H_2_O_2_ in a boiling water bath, followed by extraction with 50 mL of 1 mol·L^−1^ CH_3_COONH_4_ at room temperature for 16 h. The supernatant was collected for oxidizable Cd determination.

Step 4 (residual fraction): The final residue was sequentially treated with 1 mL of ultrapure water, 1.5 mL of HNO_3_, 0.5 mL of HCl, and 0.5 mL of HF, thoroughly mixed, and then digested in a digestion apparatus for residual Cd measurement [46].

### 4.7. Cd Content Determination

Cd concentrations in all plant and soil samples were determined using inductively coupled plasma mass spectrometry (ICP-MS, EXPEC 7200, Hangzhou Puyu Technology Development Co., Ltd., Hangzhou, China), following the protocol described by Liao et al. [21].

### 4.8. Rhizosphere Soil Microbial Community Analysis

Rhizosphere soil samples were collected following the method of Li et al. [48]. Approximately 1 g of fresh rhizosphere soil was weighed per sample, stored at −80 °C, with four biological replicates per treatment, and sent to Chengdu Luoning Biotechnology Co., Ltd. (Chengdu, China) for sequencing. Microbial community analysis was performed according to Xie et al. [49]. Total genomic DNA was extracted using the OMEGA E.Z.N.A.^®^ Soil DNA Kit (Omega Bio-Tek, Inc., Norcross, GA, USA) following the manufacturer’s instructions. gDNA was purified with the Zymo Research BIOMICS DNA Microprep Kit (Cat^#^ D4301, Irvine, CA, USA). DNA integrity was assessed by 0.8% agarose gel electrophoresis, and concentration was determined using a Tecan F200 microplate reader with PicoGreen dye (Tecan Austria GmbH, Grödig, Austria). The V4 region of the 16S rDNA was amplified using barcoded primers 515F (5′-GTGYCAGCMGCCGCGGTAA-3′) and 806R (5′-GGACTACHVGGGTWTCTAAT-3′). The 50 µL PCR reaction contained: 5 µL of 10× PCR Buffer for KOD-Plus-Neo, 5 µL of 2 mmol·L^−1^ dNTPs, 3 µL of 25 mmol·L^−1^ MgSO_4_, 1.5 µL each of 10 µmol·L^−1^ primers U515F and U806R, 1 µL of KOD-Plus-Neo DNA polymerase (1 U·µL^−1^), 2 µL of template DNA (20 ng·µL^−1^), and sterile ultrapure water to a final volume of 50 µL. PCR was performed under the following conditions: initial denaturation at 94 °C for 1 min; 30 cycles of 94 °C for 20 s, 54 °C for 30 s, and 72 °C for 30 s; and a final extension at 72 °C for 5 min. Three technical replicates were performed per sample, and equimolar amounts of PCR products in the linear amplification phase were pooled for library construction (Applied Biosystems^®^ PCR System 9700, Foster City, CA, USA). Purified PCR products were used for library preparation with the NEBNext Ultra II DNA Library Prep Kit for Illumina (NEB#E7645L, New England Biolabs, Inc., Ipswich, MA, USA). Paired-end reads were assembled using FLASH v1.2.11, and sequences were demultiplexed and barcodes trimmed using sabre based on barcode information. Raw paired-end reads were quality-filtered, denoised, and merged, and chimeras were removed using the QIIME2 dada2 (v2022.2, default parameters) denoise-paired plugin, with default parameters. Amplicon sequence variants (ASVs) were resolved using DADA2. Taxonomic assignment was performed using the SILVA database (version 138.1) with a Naive Bayes classifier. To account for uneven sequencing depth, all samples were rarefied to 25,000 sequences per sample prior to downstream analyses. α-diversity indices (Shannon, Simpson, Chao1, and PD) were calculated based on rarefied ASV tables, and differences between ecotypes were assessed using Wilcoxon rank-sum test. *β*-diversity was assessed using Principal Coordinate Analysis (PCoA) based on Bray–Curtis distance, and statistical significance was evaluated by PERMANOVA (Adonis) using the vegan package (v2.6-4) in R (v4.2.1). Differentially abundant genera between SC and SD rhizosphere soils were identified using linear discriminant analysis effect size (LEfSe) with a threshold of an LDA score > 3 and *p* < 0.05. All bioinformatics analyses were performed using the QIIME2 platform and R version 4.0.5 [50]. The raw 16S rRNA amplicon sequencing data generated in this study have been deposited in the NCBI Sequence Read Archive (SRA) under BioProject accession number PRJNA1524660 (SRA: SRP734233).

### 4.9. Isolation and Purification of Rhizosphere Bacteria from S. miltiorrhiza

Fresh rhizosphere soil samples (1.0 g) were suspended in 9 mL of sterile PBS to prepare a 10^−1^ soil dilution. The suspension was shaken at 200 rpm for 30 min at 4 °C and then allowed to stand for 30 s. Subsequently, 1.0 mL of the supernatant was transferred into a tube containing 9 mL of sterile PBS to obtain a 10^−2^ dilution. This procedure was repeated sequentially to generate a 10-fold dilution series up to 10^−7^ for subsequent analyses. Aliquots (0.2 mL) of each dilution were spread onto nutrient broth (NB) agar plates containing 10 mg·L^−1^ Cd (as CdCl_2_·2.5H_2_O). Sterile water and the final root washing solution (0.2 mL each) were spread as blank controls. Plates were incubated at 28 °C for 3~7 days, and colony morphology, color, and growth status were recorded. Single colonies were picked and inoculated into NB medium and cultured at 28 °C, and purity was verified by colony morphology observation and smear microscopy. If contamination was detected, the streak plate method was applied for purification, with three consecutive streaking rounds until microscopic examination confirmed no impurities. Purified single colonies were separately inoculated onto NB slant media or into 30% glycerol tubes. Slant cultures were incubated at 28 °C until colonies appeared, numbered, and stored at 4 °C. Glycerol stocks were stored at −80 °C for subsequent use.

### 4.10. Cd Tolerance Assay of Rhizosphere Bacteria

NB liquid medium (pH 7.0 ± 0.2) supplemented with different Cd concentrations (0, 10, 20, 40, 80, and 160 mg·L^−1^) was prepared using CdCl_2_·2.5H_2_O, with 10 mL dispensed into each test tube. Each tube was inoculated with 0.2 mL of bacterial culture and incubated at 28 °C with continuous shaking at 150 r·min^−1^ [3]. 

Bacterial growth was monitored by measuring optical density at 600 nm (A_600_) using a microplate reader (Multiskan Sky, Thermo Fisher, Waltham, MA, USA) in endpoint absorbance mode. Aliquots of 200 μL of each bacterial suspension were dispensed into 96-well clear flat bottom microplates. Sterile water and uninoculated NB medium were used as blank controls, and each treatment was performed in triplicate. Absorbance values were recorded every 2.5 h over a 30 h period. In the data preprocessing step, the instrument baseline was confirmed to have been automatically subtracted. For samples where background subtraction was incomplete, manual correction was performed using the mean absorbance of blank wells. Contaminated samples, wells with noticeable evaporation, and obvious outliers were excluded to ensure data reliability. Growth curves were constructed by plotting A_600_ against incubation time, with different Cd concentrations distinguished by color to visualize the growth dynamics of the tested strains under varying Cd stress levels.

### 4.11. Inoculation of Candidate Bacterial Strains

The experiment consisted of three categories of treatments: a control without Cd addition (CK), Cd treatment alone (Cd), and Cd treatment combined with inoculation of 16 Cd-tolerant bacterial strains (Cd + C4 to Cd + D19), respectively (Table 1). The Cd treatment was applied at 10 mg·L^−1^ (prepared as CdCl_2_·2.5H_2_O). Cd-tolerant strains were selected for reinoculation into SD. The plantlets of uniform growth and 60 days of age were transplanted into sterilized mixed substrate (nutrient soil:perlite:vermiculite = 3:0.5:0.5, v/v/v) and acclimatized for 1 week. For each treatment, 20 pots were established (one plant per pot, totaling 20 individual plants). Five plants were randomly selected for measurement of fresh weight per plant. The remaining 15 plants were randomly divided into three equal groups and pooled to serve as three biological replicates. Thus, each treatment yielded three independent pooled samples (n = 3) for subsequent analysis. Plantlets were maintained in a light-controlled growth chamber (16 h light/8 h dark, 24 °C/20 °C, 70% relative humidity). For the experimental groups, Cd treatment and bacterial inoculation were performed simultaneously on the day of transplantation. Each pot received 10 mL of bacterial suspension, which was prepared by centrifugation at 1800× *g* for 10 min and resuspension in sterile water to A_600_ = 0.6. Inoculations were repeated every 3 days, with two applications in total. The control group received an equal volume of sterile water. Sampling was performed 15 days after the first inoculation, and each treatment included three replicates [51].

### 4.12. 16S rRNA Gene Amplification and Sequencing

Single colonies of the candidate strains were picked directly from NB agar plates and resuspended in 10 μL of sterile distilled water. The full-length 16S rRNA gene was amplified using primers 27F (5′-AGAGTTTGATCMTGGCTCAG-3′) and 1492R (5′-TACGACTTAACCCCAATCGC-3′). The 25 μL PCR reaction contained 9.5 μL of ddH_2_O, 2 μL of template DNA, 0.5 μL each of forward and reverse primers (10 μmol·L^−1^), and 12.5 μL of 2× Taq MasterMix (Coolaber, China). The amplification program consisted of initial denaturation at 95 °C for 5 min; 35 cycles of 95 °C for 30 s, 60 °C for 30 s, and 72 °C for 80 s; and a final extension at 72 °C for 10 min. PCR products were verified by gel electrophoresis and sent to Chengdu Tsingke Biotechnology Co., Ltd. for paired end sequencing. The obtained sequences were compared using BLAST against the NCBI database (https://www.ncbi.nlm.nih.gov/). A phylogenetic tree was constructed using MEGA 12 with the Neighbor-Joining (NJ) method (see Supplementary Table S1 for relevant bacterial species and their accession numbers), and bootstrap analysis was performed with 1000 replicates.

### 4.13. Cd Adsorption Kinetics of Rhizosphere Bacteria

The tested strains were inoculated into 30 mL of nutrient broth (NB) liquid medium and cultured at 28 °C with shaking at 160 r·min^−1^ for 14 h to obtain the seed cultures. Subsequently, 250 μL of each seed culture was transferred into flasks containing 75 mL of NB medium supplemented with 10 mg·L^−1^ Cd (as CdCl_2_·2.5H_2_O). The cultures were incubated under the same conditions for 12, 24, 36, 48, and 60 h, with three independent biological replicates per time point. At each designated sampling time, an aliquot of the bacterial suspension was centrifuged at 1800× *g* for 10 min at 4 °C. The supernatant was collected as the free Cd^2+^ fraction. The resulting pellet was resuspended in 20 mmol·L^−1^ EDTA solution (pH 7.0) and shaken for 2 h to desorb surface bound Cd, including Cd associated with cell walls and extracellular polymeric substances. After centrifugation at 9500× *g* for 10 min, the supernatant was collected as the extracellular Cd fraction. The residual pellet was washed three times with ultrapure water to remove residual EDTA, and the Cd content of the washed pellet was designated as the intracellularly accumulated Cd fraction [52]. All three Cd fractions were digested and determined by the method detailed in Section 4.7.

### 4.14. Isolation and Determination of Cell Wall Polysaccharide Fractions

Root samples (1 g each) of *S. miltiorrhiza* seedlings from the CK, Cd, and Cd + B treatments (all at 10 mg·L^−1^ CdCl_2_·2.5H_2_O; bacterial suspension A_600_ = 0.6, resuspended in sterile water) were ground and soaked three times in 75% ice-cold ethanol, followed by centrifugation at 6000× *g* for 10 min. The pellet was sequentially washed with ice-cold acetone, ice-cold methanol chloroform (1:1, v/v), and ice-cold methanol. The resulting cell wall samples were freeze-dried and stored at 4 °C until further use. Three biological replicates and four technical replicates were performed for each treatment. Separation, extraction, and quantification of pectin and hemicellulose were performed according to Liao et al. [21], and Cd content in pectin and hemicellulose fractions was determined as described in Section 4.7.

### 4.15. Subcellular Distribution of Cd in Roots

Subcellular fractionation of Cd in roots was performed following the method of Liao et al. [21] using a stepwise chemical extraction procedure to separate cell wall, organelle, soluble, and nuclear/residual fractions. The extracted cell wall and organelle fractions were dried in an oven at 60 °C. Cd concentrations in all fractions were determined using the method described in Section 4.7, and Cd contents were calculated accordingly.

### 4.16. Evaluation of Pharmacological Quality

SD plantlets were cultivated in soil collected from a Cd-contaminated area (approximately 104.1° E, 31.2° N) in a city of Sichuan Province, with a Cd content of 1.3 mg·kg^−1^. Plants were grown in plastic pots (42 cm × 40 cm) containing 8 kg of Cd-contaminated soil per pot, with five plantlets per pot and three replicates. The following treatments were established: control (MoCK), inoculation with strain C4 (C4 group), and inoculation with strain D9 (D9 group). After 4 months of cultivation, *S. miltiorrhiza* roots were harvested, oven-dried, ground, and passed through a No. 3 sieve. For tanshinone determination, 0.3 g of powder was extracted with 50 mL of methanol by ultrasonication for 30 min and filtered through a 0.22 μm membrane. For salvianolic acid B determination, 0.15 g of powder was extracted with 50 mL of 80% methanol by ultrasonication for 30 min and filtered through a 0.22 μm membrane. The contents of tanshinone I, tanshinone IIA, cryptotanshinone, and salvianolic acid B were determined by high-performance liquid chromatography (ZORBAX SB-C18, HPLC, Agilent 1260, Santa Clara, CA, USA) following the method described in the *Chinese Pharmacopoeia (2025 edition)* [53].

### 4.17. Statistical Analysis

Data were organized and processed using Excel 2019. All results are presented as mean ± standard deviation (SD), with three independent replicates per treatment. Differences among treatments were assessed using one-way analysis of variance (ANOVA) and two-way ANOVA, with statistical significance set at *p* < 0.05. Post hoc mean comparisons were performed using Duncan’s multiple-range test or Tukey’s honestly significant difference (HSD) test in SPSS 27.

## 5. Conclusions

This study successfully isolated two functionally divergent *Bacillus* strains, *Bacillus* sp. C4 from low-Cd-accumulating *S. miltiorrhiza* and *Bacillus* sp. D9 from its high-Cd-accumulating counterpart. Strain C4 effectively reduces Cd accumulation, promotes plant growth, and enhances pharmacopeia constituents’ contents, whereas strain D9 significantly facilitates Cd uptake and biomass production. A working model is proposed in which C4 restricts Cd uptake through a combination of Cd sequestration by bacterial cells, rhizosphere alkalinization, and reduced pectin and hemicellulose contents in root cell walls, whereas D9 promotes Cd accumulation through rhizosphere acidification, hemicellulose enrichment, and altered root morphology. To our knowledge, this is the first report of congeneric rhizobacteria with opposing regulatory effects on Cd accumulation in the medicinal plant *S. miltiorrhiza*, and it provides a conceptual framework—integrating physicochemical and biological processes—for understanding rhizobacterial modulation of heavy metal phytoavailability. Collectively, these findings offer candidate microbial resources with the potential for developing biofertilizers to enhance the quality and safety of *S. miltiorrhiza* grown in Cd-contaminated soils.

## Figures and Tables

**Table 1:** Effects of reinoculation with different Cd-tolerant bacterial strains on fresh weight and Cd accumulation in *S. miltiorrhiza*.

Treatments	Fresh Weight	Cd Content in the Shoot (μg g^−1^)	Cd Content in the Root (μg g^−1^)	Cd Accumulation in the Shoot (μg plant^−1^)	Cd Accumulation in the Root (μg plant^−1^)	Total Cd Accumulation in the Plant (μg plant^−1^)
Cd	1.004 ± 0.14 de	13.082 ± 0.15 f	128.708 ± 1.01 ij	9.190 ± 0.12 ij	41.538 ± 0.50 l	50.728 ± 0.51 k
Cd + C4	1.168 ± 0.20 de	10.388 ± 0.08 gh	112.151 ± 1.30 j	7.308 ± 0.57 kl	33.087 ± 0.33 m	40.395 ± 0.68 l
Cd + C6	0.92 + 0.08 e	21.199 ± 1.37 b	208.559 ± 12.14 g	13.673 ± 0.88 fg	57.562 ± 3.34 k	71.235 ± 4.22 ij
Cd + C7	1.51 ± 0.34 bc	16.118 ± 0.65 e	303.887 ± 28.29 e	18.488 ± 0.74 bc	110.007 ± 10.24 e	128.495 ± 9.50 e
Cd + C9	1.052 ± 0.14 de	7.930 ± 0.11 i	133.079 ± 2.01 i	6.397 ± 0.47 l	42.944 ± 0.72 l	49.341 ± 1.00 k
Cd + C11	1.7 ± 0.13 ab	11.922 ± 0.07 fg	241.634 ± 1.11 f	15.403 ± 0.37 de	71.240 ± 0.51 gh	86.643 ± 0.81 gh
Cd + C16	1.91 ± 0.05 a	8.814 ± 0.11 hi	114.517 ± 0.25 ij	12.195 ± 0.27 efg	57.35 ± 0.34 k	69.545 ± 0.55 j
Cd + D1	1.773 ± 0.38 ab	8.385 ± 0.60 i	429.580 ± 0.90 c	12.075 ± 0.86 gh	143.05 ± 0.30 d	155.125 ± 0.56 d
Cd + D5	1.896 ± 0.34 a	17.247 ± 0.47 de	507.421 ± 2.20 b	16.827 ± 0.22 cd	182.164 ± 0.79 b	198.991 ± 1.01 b
Cd + D6	1.122 ± 0.25 de	17.05 ± 2.19 de	356.358 ± 40.75 d	15.550 ± 1.99 d	74.478 ±8.52 g	90.028 ± 10.51 g
Cd + D7	1.08 ± 0.22 de	18.259 ± 2.09 d	290.110 ± 5.27 e	13.822 ± 1.59 ef	94.286 ± 1.71 f	108.108 ± 0.12 f
Cd + D9	1.52 ± 0.40 bc	23.276 ± 0.86 a	617.328 ± 2.63 a	23.094 ± 0.42 a	224.708 ± 0.96 a	247.802 ± 1.38 a
Cd + D10	1.266 ± 0.19 cd	9.037 ± 0.16 hi	215.975 ± 2.38 g	8.642 ± 0.22 jk	66.737 ± 0.74 hi	75.379 ± 0.67 i
Cd + D12	1.28 ± 0.17 cd	24.562 ± 3.82 a	501.812 ± 4.02 b	23.653 ± 3.68 a	158.071 ± 1.27 c	181.724 ± 4.95 c
Cd + D13	1.266 ± 0.28 cd	11.135 ± 0.14 g	111.768 ± 0.12 i	9.688 ± 0.45 ij	35.926 ± 0.27 m	45.614 ± 0.18 kl
Cd + D15	1.716 ± 0.21 ab	8.604 ± 0.22 i	157.831 ± 1.32 h	10.570 ± 0.52 hi	60.789 ± 0.30 jk	71.359 ± 0.50 ij
Cd + D19	1.526 ± 0.24 bc	17.069 ± 0.07 de	176.238 ± 2.40 h	19.235 ± 0.60 b	62.409 ± 1.25 ij	81.644 ± 1.82 h

Note: Data are presented as means ± standard deviation (SD) (fresh weight: n = 5; Cd content and accumulation: n = 3). Different letters indicate significant differences among treatments for fresh weight, Cd content, and Cd accumulation, as determined by one-way ANOVA followed by Duncan’s multiple comparison test (*p* < 0.05).

## Data Availability

The original contributions presented in this study are included in the article/Supplementary Material. Further inquiries can be directed to the corresponding author.

## Supplementary Materials

The following supporting information can be downloaded at https://www.mdpi.com/article/10.3390/plants15182858/s1.
