# Supplementary material for: Screening of Rhizobacteria Capable of Modulating Cd Accumulation and Enhancing Pharmacopoeial Constituents in Salvia miltiorrhiza
Source: Plants (Basel). 2026 Sep 18;15(18):2858. doi: 10.3390/plants15182858 (PMC13610631; doi:10.3390/plants15182858)
Supplement: Supplementary file 1 [file plants-15-02858-s001.zip › Supplementary figure.pdf]

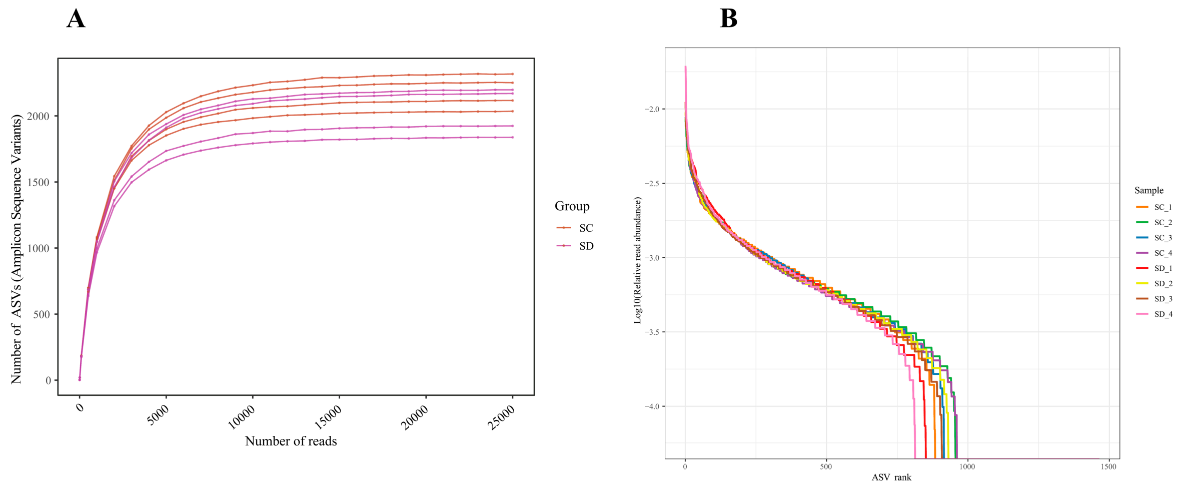

Supplementary Figure S1: Quality Assessment of 16S rRNA High throughput Sequencing Data.

(A) Rarefaction curves of SC and SD samples; (B) Rank abundance curves of ASVs. SD, high Cd accumulating ecotype of *S. miltiorrhiza*; SC, low Cd accumulating ecotype.

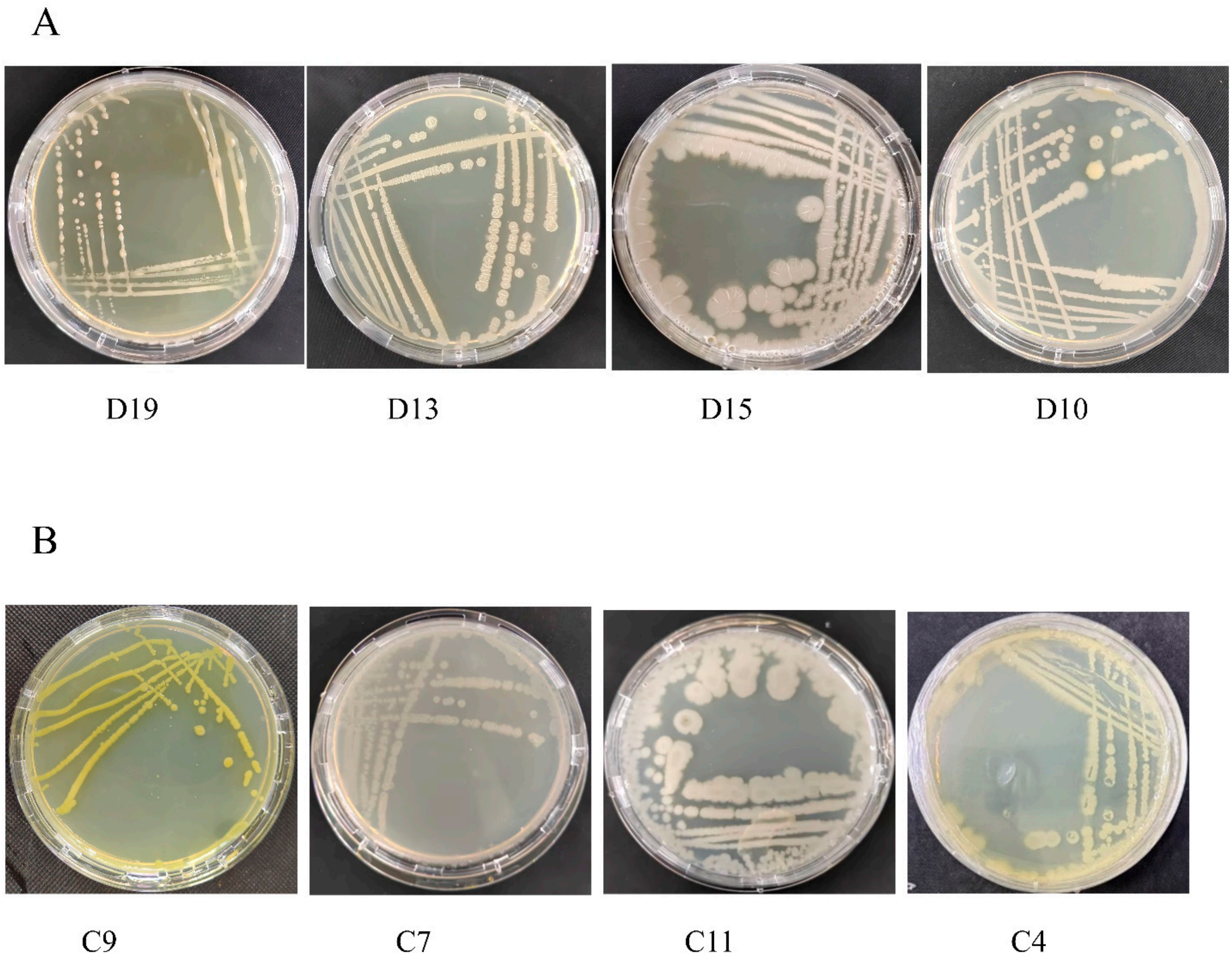

Supplementary Figure S2: Isolation of Cd-tolerant Bacteria on NB Agar Medium Supplemented with 10 $mg\cdot L^{-1}$ Cd.

(A) Single colony morphology of Cd tolerant strains (D19, D13, D15, D10) isolated from the rhizosphere of SD.

(B) Single colony morphology of Cd tolerant strains (C9, C7, C11, C4) isolated from the rhizosphere of SC.

All strains were cultured on NB agar medium by streaking, and exhibited distinct colony morphologies in terms of color, size, and elevation.

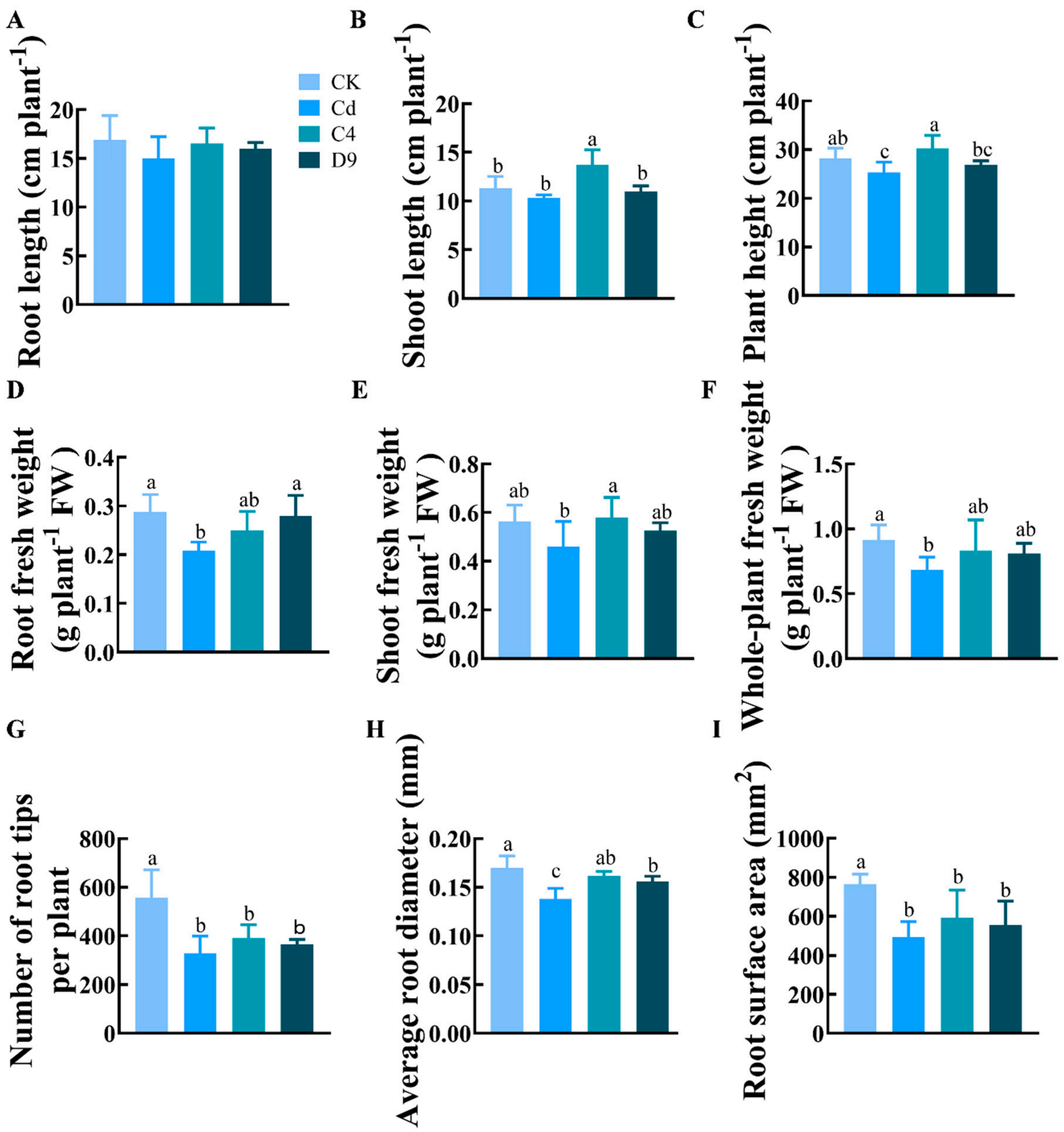

Supplementary Figure S3: Growth related parameters of the high Cd accumulating ecotype (SD) of *S. miltiorrhiza* following inoculation with strains D9 and C4.

(A) Root length; (B) Shoot height; (C) Total plant height; (D) Root fresh weight; (E) Shoot fresh weight; (F) Whole plant fresh weight; (G) Number of root tips; (H) Average root diameter; (I) Root surface area. CK, 0 mg·L⁻¹ Cd (noninoculated control); Cd, 10 mg·L⁻¹ Cd (noninoculated); C4, 10 mg·L⁻¹ Cd + *Bacillus* sp. C4; D9, 10 mg·L⁻¹ Cd + *Bacillus* sp. D9. Data are presented as means ± SD (n = 5). Different letters indicate significant differences among treatments, as determined by one-way ANOVA followed by Duncan's multiple range test ($p < 0.05$).
