# Supplementary material for: Screening of Rhizobacteria Capable of Modulating Cd Accumulation and Enhancing Pharmacopoeial Constituents in Salvia miltiorrhiza
Source: Plants (Basel). 2026 Sep 18;15(18):2858. doi: 10.3390/plants15182858 (PMC13610631; doi:10.3390/plants15182858)
Supplement: Supplementary file 1 [file plants-15-02858-s001.zip › Supplementary table.pdf]

Supplementary Table S1: Strains Used for Phylogenetic Analysis and Their GenBank Accession Numbers.

**Species**	**Login ID**	**Species**	**Login ID**
Bacillus inaquosorum	NR 104873	*Metabacillus bambusae*	NR 180924
Bacillus cabrialesii	MK462260	*Bacillus litoralis*	KC414705
Bacillus cabrialesii	NR 180419	*Metabacillus schmidteae*	NR 179484
*Bacillus mojavensi*s	NR 024693	*Metabacillus niabensis*	NR 043334
Bacillus atrophaeus	NR 112723	*Fredinandcohnia*	OM791795
Bacillus licheniformis	MN396732	*Bacillus timonensis*	NR 133024
Bacillus salacetis	NR 179253	*Bacillus nitroreducens*	NR 178876
Rossellomorea arthrocnemi	PX841745	*Bacillus nitroreducens*	KT725236
Bacillus oryzaecorticis	MZ27629	*Bacillus suaedaesalsae*	NR 181631
Bacillus horneckiae	FR749913	*Bacillus mesophilus*	NR 149175
Bacillus salipaludis	NR 180481	*Sutcliffiella catenulata*	NR 179565
Bacillus fengqiuensis	NR 133973	*Sutcliffiella cohnii*	NR 113776
Bacillus thaonhiensis	NR 125615	*Sutcliffiella horikoshii*	NR 119070
Sutcliffiella halmapala	NR 026144	*Bacillus aequororis*	NR 181752
Bacillus aequororis	NR 178580	*Bacillus aequororis*	MZ276295
Pseudoneobacillus rhizosphaerae	NR 181873	*Gottfriedia endophytica*	NR 181575
Gottfriedia solisilvae	NR 159143	*Gottfriedia solisilvae*	NR 159143
Bacillus solisilvae	MT 759868	*Bacillus yunxiaonensis*	NR 199426
Bacillus mycoides	NR 115993	*Bacillus pseudomycoides*	NR 114422
Bacillus mycoides	OR841474	*Bacillus pseudomycoides*	PV569768
Bacillus thuringiensis	CP021061	*Bacillus wiedmannii*	PP068841
